# Appearance of Recalcitrant Dissolved Black Carbon and Dissolved Organic Sulfur in River Waters Following Wildfire Events

**DOI:** 10.1021/acs.est.4c00492

**Published:** 2024-04-10

**Authors:** Yanghui Xu, Xintu Wang, Qin Ou, Zhongbo Zhou, Jan Peter van der Hoek, Gang Liu

**Affiliations:** † Key Laboratory of Drinking Water Science and Technology, Research Centre for Eco-Environmental Sciences, Chinese Academy of Sciences, Beijing 100085, China; ‡ Section of Sanitary Engineering, Department of Water Management, Faculty of Civil Engineering and Geosciences, Delft University of Technology, Stevinweg 1, 2628 CN Delft, The Netherlands; § College of Environmental Science and Engineering, Guilin University of Technology, Guangxi 541004, China; ∥ College of Resources and Environment, Southwest University, Chongqing 400715, China; ⊥ Waternet, Department Research & Innovation, P.O. Box 94370, 1090 GJ Amsterdam, The Netherlands; # University of Chinese Academy of Sciences, Beijing 101408, China

**Keywords:** wildfires, dissolved organic matter, dissolved black carbon, dissolved black nitrogen, sulfur-containing proteins

## Abstract

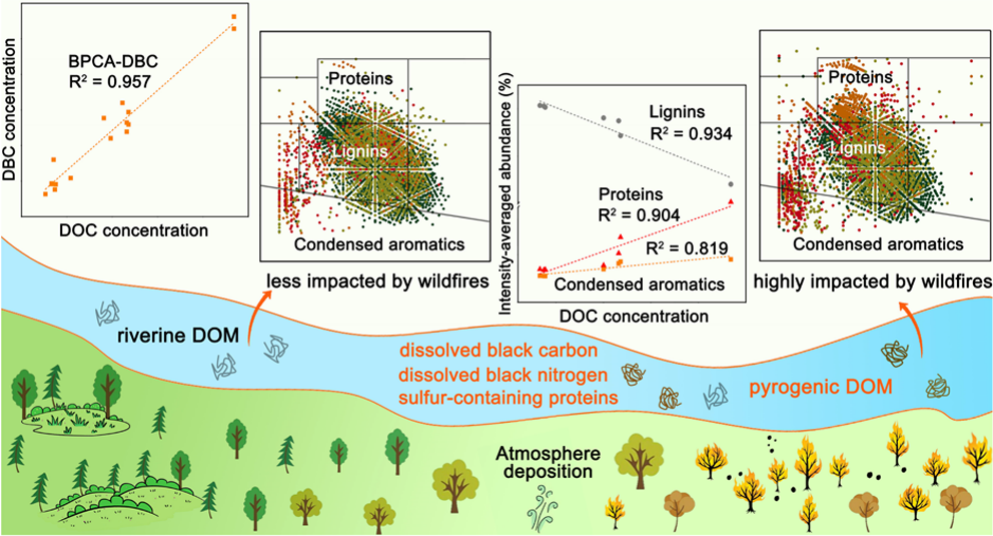

Increasing wildfire frequency, a consequence of global climate change, releases incomplete combustion byproducts such as aquatic pyrogenic dissolved organic matter (DOM) and black carbon (DBC) into waters, posing a threat to water security. In August 2022, a series of severe wildfires occurred in Chongqing, China. Samples from seven locations along the Yangtze and Jialing Rivers revealed DBC, quantified by the benzene poly(carboxylic acid) (BPCA) method, comprising 9.5–19.2% of dissolved organic carbon (DOC). High concentrations of BPCA-DBC with significant polycondensation were detected near wildfire areas, likely due to atmospheric deposition driven by wind. Furthermore, Fourier transform ion cyclotron resonance mass spectrometry (FT-ICR-MS) revealed that wildfires were associated with an increase in condensed aromatics, proteins, and unsaturated hydrocarbons, along with a decrease in lignins. The condensed aromatics primarily consisted of dissolved black nitrogen (DBN), contributing to abundant high-nitrogen-containing compounds in locations highly affected by wildfires. Meanwhile, wildfires potentially induced the input of recalcitrant sulfur-containing protein-like compounds, characterized by high oxidation, aliphatic nature, saturation, and low aromaticity. Overall, this study revealed the appearance of recalcitrant DBC and dissolved organic sulfur in river waters following wildfire events, offering novel insights into the potential impacts of wildfires on water quality and environmental biogeochemistry.

## Abstract 2

Atmospheric ash deposition immediately following wildfires contributed to the appearance of recalcitrant dissolved black carbon and black nitrogen, as well as sulfur-containing proteins, in river waters.

## Introduction

The rising frequency and severity of wildfires, closely tied to climate change, are expected to further escalate as climate change persists.^1,2^ Wildfires emit significant amounts of carbon into the atmosphere in the form of CO_2_ due to the combustion of biomass.^1^ Wildfires also produce substantial quantities of pyrogenic organic matter (OM) during the incomplete combustion of vegetation and soil organic material.^3,4^ Rivers originating in fire-affected areas can undergo significant alterations during and after burn events, impacting water quality and freshwater ecosystem productivity.^5,6^ Particularly, the export of pyrogenic OM in the form of dissolved organic matter (DOM) into rivers has the potential to adversely affect water quality and can influence carbon biogeochemical cycling and aquatic ecosystem functioning.^7,8^ Dissolved black carbon (DBC), the largest known refractory DOM pool in aquatic systems, has arisen great attention for its roles in regulating greenhouse gas emissions and stabilizing DOM.^9,10^

The relationship between wildfires and riverine DOM/DBC has been a research hotspot, but it remains not well understood. Ding et al. reported that there was no correlation between recent fire activities and in-stream dissolved organic carbon (DOC) and DBC concentrations.^11,12^ However, Dittmar et al. observed continued export of riverine DBC and DOC each year in the rainy season after major fire activity.^7^ The concentrations of DOC in streamwater were also identified to be primarily driven by local hydrology during the first storm event occurring 1 month after the fire.^13^ Generally, most studies have investigated the correlation between wildfires and riverine DOM/DBC over an extended period, occurring several months or years after the wildfires took place.^7,14^ Environmental drivers, especially hydrology during rain and storm events are recognized as significant factors determining the postfire export of DOC and DBC.^10,11,15,16^ However, in scenarios where there is no rainfall, the immediate impacts of wildfires on aquatic DOM and DBC within a few days postfire remain poorly understood. Apart from soil infiltration and surface runoff,^17^ the atmosphere dry deposition of aerosols is recognized as another significant source of DBC in watersheds.^9,18,19^ Therefore, it is reasonable to assume that wildfires can have immediate impacts on riverine DOM/DBC via atmospheric deposition of ash.

The aim of this article was to investigate the potential impacts of wind/air deposition of ash on riverine DOM and DBC in the adjacent rivers immediately after wildfires. We collected seven samples a few days after the wildfires from locations near the wildfire-affected areas of the Yangtze and Jialing Rivers located in Chongqing. The riverine DOM and DBC were quantitatively assessed using total organic carbon (TOC) and benzene poly(carboxylic acid) (BPCA) methods, respectively. Additionally, Fourier transform ion cyclotron resonance mass spectrometry (FT-ICR-MS) was employed to characterize the molecular composition of both DOM and DBC. By combining BPCA and FT-ICR-MS analysis, this work aimed to investigate the effects of wildfires on both the quantity and quality of aquatic DOM and DBC.

## Materials and Methods

### Sites and Sampling

Chongqing, situated in southwestern China, covers a total area of 82,400 km^2^. The topography in this region is characterized by hills and mountains, and it is intersected by two major rivers, the Yangtze River and the Jialing River, at the center (Figure S1). Since July 2022, Chongqing has been facing an extended period of severe drought marked by minimal precipitation and consistently high temperatures. There have been several forest fires in Chongqing since August 9. On the night of August 17, a forest fire initiated in the mountains and forests within the Fuling District of Chongqing. The fire swiftly propagated to other districts, resulting in a series of fires that endured for 9 days until all wildfires were fully extinguished on August 26.

**Figure 1:**
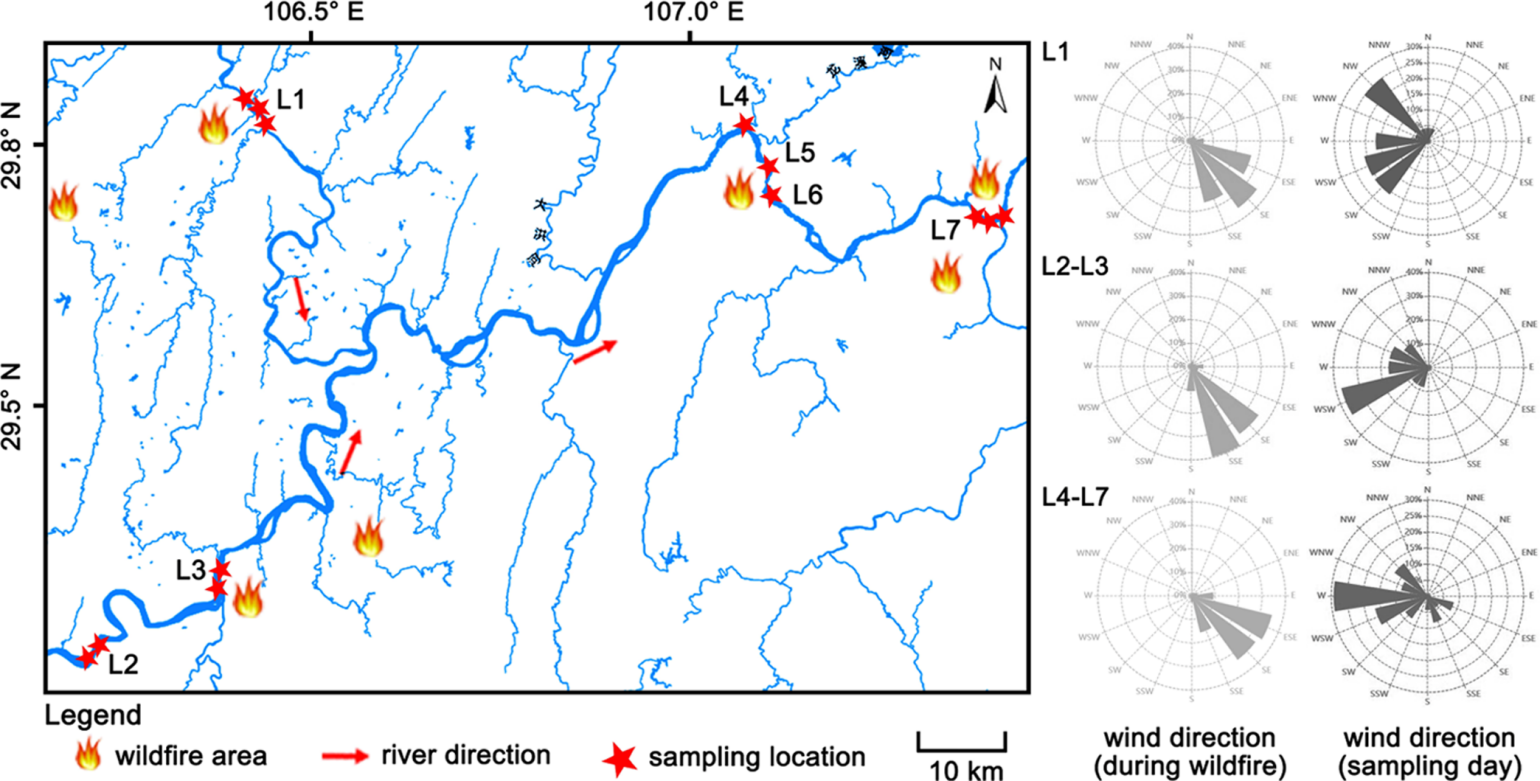
Location of the study area, different wildfire areas, and sampling sites.

On August 29, water samples were collected from seven locations along the Yangtze River and the Jialing River (Figures 1 and S2). Table S1 provides information about the sampling locations. Location L2, situated upstream of the main river and far from the nearest wildfire site, should not have been affected by the wildfire event. However, at location L7, burned areas were observed along the riverbank (Figure S2), although no visual differences were observed among the other locations. Notably, there was a notable shift in wind direction from the predominant southeast wind during wildfires to west and west-north winds on the sampling day (Figure 1). The selection of these locations aimed to be as close as possible to the wildfire areas. Replicate samples were taken at approximately 1 km intervals, but some locations had only one or two samples due to geographical constraints. Each water sample (5 L) was directly collected using a 5 L glass bottle from the surface at a depth of 0–20 cm and sent back to the laboratory within 1 day. Water quality parameters including pH, dissolved oxygen (DO), electrical conductivity (EC), oxidation–reduction potential (ORP), and turbidity (TB) were measured in situ using a multiparameter controller on the sampling day. After this, the water samples were subjected to filtration using 0.45 μm membrane filters (Millipore) and then stored at 4 °C for subsequent analyses. In 3 days, DOC was analyzed using a TOC analyzer, and NH_4_^+^ and NO_3_^–^ were measured using flow injection analysis. The partial filtrate (2 L) was acidified to pH 2 using HCl to inhibit any microorganic activity before DOM extraction using solid-phase extraction.

### Extraction of DOM Using Solid-Phase Extraction

DOM was extracted using PPL-resin-based solid-phase extraction (500 mg, 6 mL, Agilent) following the procedure of Dittmar et al.^20^ DOM extraction efficiency of PPL cartridges is typically higher than 60%.^21−23^ The PPL cartridges were initially rinsed with acidified water and activated with methanol. The filtered water samples were allowed to pass through the cartridge at a flow rate of 5 mL min^–1^. To remove salts, the cartridges were rinsed with 50 mL of acidified water before being dried under N_2_. Subsequently, the absorbed DOM was eluted with 10 mL of methanol. The eluted samples were blow-dried with N_2_, redissolved in a 1 mL methanol solution, and stored in the dark at −20 °C. For BPCA analysis, each sample from the seven locations was measured. Individual samples from L4, L5, and L6, as well as the second sample from L1, L2, L3, and L7, were employed for FT-ICR-MS analysis.

### Quantification of DBC Based on the BPCA Method

DBC concentration was quantified with the BPCA method.^24,25^ 0.2 mL of concentrated DOM sample was transferred into a 2 mL Teflon digestion tube. After drying with N_2_, the samples were digested with 0.5 mL 65% nitric acid at 170 °C for 9 h. After oxidation, the remaining nitric acid and water were dried under high-purity N_2_ at 50 °C. After drying, 1 mL of methanol was added to redissolve the samples, with 10 μg of biphenyl-2′2-dicarboxylic acid as an internal standard.

BPCAs were analyzed using an Agilent 1260 Infinity HPLC system equipped with a photodiode array detector. Samples were eluted at a flow rate of 1 mL/min following gradients from 90% mobile phase A (0.1% phosphoric acid) to 90% mobile phase B (methanol) over 35 min with a Luna phenylhexyl column (5 μm, 4.6 × 250 mm^2^). The detailed method is provided in Table S2. The quantification of the BPCAs was carried out with absorbance at 235 nm using the calibration curve of the BPCA standard mixture (Table S3). The standard mixture of the BPCAs was prepared with commercially available BPCAs (1,2,3-B3CA; 1,2,4-B3CA; 1,3,5-B3CA; 1,2,4,5-B4CA; B5CA; B6CA). The calibration curve of 1,2,4,5-B4CA was used to quantify 1,2,3,4-B4CA and 1,2,3,5-B4CA.^26^ Moreover, the concentrations of BPCAs were converted to DBC concentrations using an equation proposed by Dittmar.^27^

### Ultrahigh-Resolution Mass Spectrometry

A Bruker Solarix 15 T FT-ICR-MS instrument mass spectrometer equipped with a 9.4 T superconducting magnet and an Apollo II electrospray ion source (ESI) was used for mass spectral analysis. DOM samples were injected into an ESI source in negative mode at a rate of 2 μL/min, and each run included 500 scans from 150 to 2000 Da. A total of 128 scans with 2 M word sizes were accumulated to improve the signal-to-noise (S/N) ratio. Mass peaks in the *M*_w_ range of 200–800 with S/N greater than 4 and an error value lower than 1.0 ppm were used to assign the correct molecular formula. A molecular formula calculator generated matching formulas according to elemental combinations of ^12^C_0–50_, ^1^H_0–150_, ^16^O_0–50_, ^14^N_0–4_, and ^32^S_0–2_. All elemental formulas must meet the basic chemical criteria: (1) 2 ≤ H ≤ (2C + 2); (2) 0 ≤ O ≤ (C + 2); (3) O/C < 1.2 and 0.33 ≤ H/C < 2.3; (4) N/C < 0.5 and S/C < 0.2;

The aromaticity index (AI_mod_) was calculated directly from molecular formulas as (1 + C-0.5O–S-0.5N)/(C-0.5O–S–N). AI_mod_ values ≥ 0.5 and ≥ 0.67 were identified as aromatic and condensed aromatic structures (DBC), respectively.^28^ Furthermore, DOM molecules can be categorized into seven groups: (1) condensed aromatics (CAs; AI_mod_ ≥ 0.67); (2) tannins (0.67 ≤ O/C < 1.2, H/C< 1.5, AI_mod_ < 0.67); (3) lignins (0.1 ≤ O/C < 0.67, H/C < 1.5, AI_mod_ < 0.67); (4) unsaturated hydrocarbons (UHs; O/C < 0.1, H/C < 1.5, AI_mod_ < 0.67); (5) carbohydrates (0.67 ≤ O/C < 1.2, 1.5 ≤ H/C < 2.3); (6) proteins (0.2 ≤ O/C < 0.67, 1.5 ≤ H/C < 2.3); (7) lipids (0.67 ≤ O/C < 1.2, 1.5 ≤ H/C < 2.3); (6) proteins (O/C < 0.2, 1.5 ≤ H/C < 2).^22,29^ The intensity-averaged abundance (wa) parameters such as double-bond equivalence (DBE_wa_), molecular weight (Mw_wa_), H/C_wa_, and O/C_wa_ were calculated based on the assigned formulas of each DOM molecule. Additional details are available in Text S1.

## Results and Discussion

### DBC Concentrations and BPCA Composition

Table 1 shows the concentrations of DOC and DBC at sampled sites. The DOC concentrations of samples collected for the seven sites ranged from 1.66 to 5.68 mg/L. The DBC concentrations observed in the studied rivers were 0.17 to 1.09 mg/L, consistent with the global range of riverine DBC concentrations (0.002 to 2.77 mg/L).^30,31^ The DBC/DOC ratio in the studied rivers was further calculated and fell within the range of 9.5 to 19.2% (Table 1). Typically, the reported DBC proportions in diverse rivers have exhibited a wide range spanning from 0.1 to 17.5%,^30,31^ but the majority remained below 10% (Table 2). In a comprehensive investigation of China’s major rivers, including the Yangtze, Yellow, Pearl, and Heilongjiang rivers, the DBC concentrations were comparatively lower (0.03–0.34 mg/L).^32^ Notably, the Heilongjiang River stood out with the highest DOC concentrations (5.62–12.86 mg/L) but much lower DBC concentrations (0.07–0.09 mg/L) compared to other rivers.^32^ It reflected a relatively lower input of DBC in the Heilongjiang region, attributed to its less populated and colder northern climate with fewer landscape fires, in contrast to the more densely populated and warmer southern regions that experience more biomass burning.^32^ At location L2 that was obtained from upstream of the main river (Yangtze River) and far from the nearest wildfire site (21 km), the lowest DBC concentration (0.17 mg/L) was detected, indicating that it might be nonimpacted. At locations L1, L3, L5, and L6, where higher levels of DOC were recorded, correspondingly, elevated concentrations of DBC were also observed. These specific sites were also situated in close proximity to points of wildfire occurrence (2.5–5.2 km), suggesting a possible impact from the wildfire events. Typically, the hydrology during rain and storm events was considered the primary factor driving the input of DBC from wildfire-affected lands.^14,15,33^ However, due to the absence of rainfall between the wildfire event and the sampling day (Figure S3), the hydrology was not the reason for riverine DBC input. Meanwhile, the daily average concentrations of atmospheric OC and BC experienced multiple peaks on wildfire days, followed by a decline to their lowest levels on August 27th, after the wildfires (Figure S4).^25,34^ Thus, the atmospheric ash deposition might be a significant contributor to riverine DBC. Notably, all samples were collected 3 days postfire, which may have resulted in changes in concentrations or compositions during the initial days via potential biogeochemical processes.^35−37^ Especially, despite its proximity to two wildfire areas (1.1–2.3 km), L7 exhibited relatively low levels of DOC and DBC. Unlike other locations (e.g., L1, L5, and L6) located downwind on the sampling day, L7 was parallel to the prevailing wind direction (Figure 1). Consequently, the wind patterns on the sampling day might have facilitated ash deposition in adjacent rivers. Therefore, it is reasonable to infer that locations L2, L4, and L7 were nonimpacted or less impacted by wildfires, while L1, L3, L5, and L6 were highly impacted, potentially due to atmospheric deposition of ash facilitated by wind.

**Table 1:** DOC, DBC, DBC/DOC, (B6 + B5)/(B4 + B3) and Basic Quality Parameters at Different Locations

location	DOC (mg/L)	DBC (mg/L)	DBC/DOC	(B6 + B5)/(B4 + B3)	pH	ORP (mV)	NH_4_^+^ (mg/L)	NO_3_^–^ (mg/L)	EC (μs/cm)	DO (mg/L)	TB (NTU)
L6	5.68	1.09	0.192	7.54	8.05	318	0.077	0.890	331	8.7	7.8
L3	3.36	0.55	0.164	4.90	7.77	89.2	1.824	3.355	347	8.2	8.8
L5	3.32	0.50	0.151	5.66	7.97	348	0.106	0.503	337	8.6	10.6
L1	3.00	0.54	0.181	7.51	7.74	218	0.066	1.329	299	7.4	4.0
L7	1.77	0.22	0.125	0.83	7.77	348	0.105	1.422	354	8.4	7.6
L4	1.66	0.18	0.111	2.80	8.04	333	0.052	0.889	334	8.6	3.9
L2	1.75	0.17	0.095	2.89	7.94	124	0.052	0.890	330	8.2	11.5

**Table 2:** 

sampling location	DOC (mg/L)	DBC (mg/L)	DBC/DOC (%)	references
Poudre river	1.56–8.41	0.05–0.34	3.0–6.6	(43)
Jiulong river	7–18.5	0.06–0.27	0.4–3.2	(47)
Biomes in North America	0.77–4.01	0.023–0.049	0.4–3.4	(12)
North America	1.40–2.50	0.04–0.11	2.5–3.8	(11)
Apalachicola National Estuarine Research Reserve	1.04–7.29	0.007–0.49	0.7–8.0	(7)
Global rivers	0.67–14.77	0.02–2.06	2.1–15.2	(31)
Global rivers	−	0.002–2.77	0.1–17.5	(30)
Chongqing Jialing and Yangtze River	1.66–5.68	0.17–1.09	9.5–19.2	this study

**Table 3:** Molecular Parameters of the DOM from Different Locations

location	formula	*M*_w,wa_	C_wa_	H_wa_	O_wa_	N_wa_	S_wa_	H/C_wa_	O/C_wa_	DBE_wa_	(AI_mod_)_wa_	NOSC_wa_
L6	5262	439.914	21.15	24.99	9.42	0.210	0.228	1.275	0.464	9.263	0.226	–0.292
L3	5171	412.657	20.12	22.68	8.46	0.286	0.283	1.205	0.445	9.420	0.280	–0.234
L5	5161	416.544	19.82	22.23	9.27	0.259	0.140	1.182	0.484	9.332	0.288	–0.159
L1	5007	452.213	21.42	23.47	10.33	0.261	0.082	1.150	0.498	10.309	0.301	–0.109
L7	4675	456.714	21.57	23.62	10.58	0.189	0.070	1.144	0.500	10.353	0.304	–0.108
L4	4714	454.941	21.51	23.52	10.54	0.180	0.066	1.143	0.499	10.338	0.305	–0.110
L2	4683	457.604	21.58	23.49	10.66	0.176	0.061	1.138	0.503	10.419	0.307	–0.098

**Figure 2:**
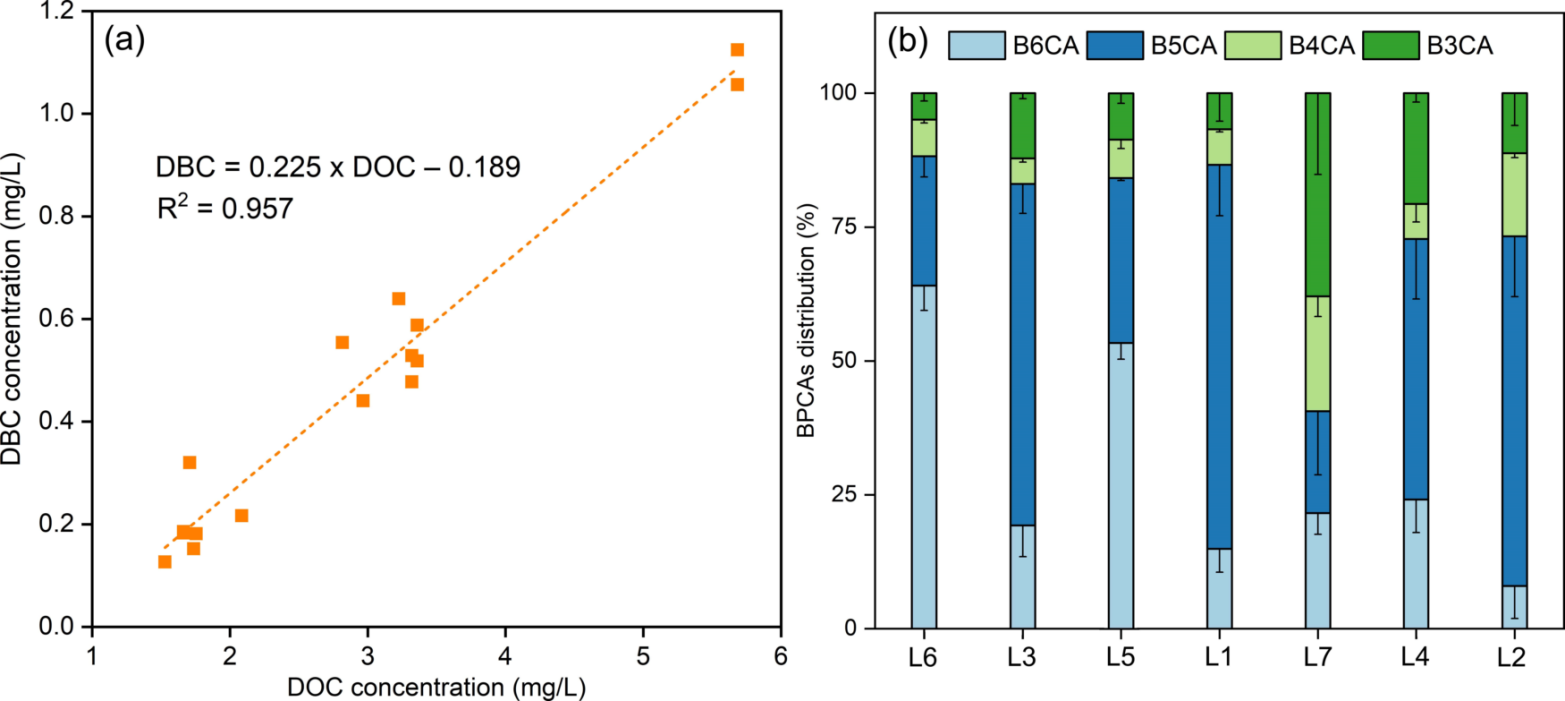
DOC and DBC correlation (a) and BPCA distributions of BPCA-DBC(b) from different locations.

Although noticeable variability was observed across all sites, a strong correlation existed between DBC and DOC concentrations, as evidenced by a high correlation coefficient (*R*^2^ = 0.957, *p* < 0.05) (Figure 2a). A high correlation between DOC and DBC in global rivers has also been documented, indicating that the mobilization of riverine DBC and DOC was mechanistically coupled.^30^ A diminished correlation between DBC and DOC concentrations has been observed in some rivers, including the Amazon River,^38^ and China’s major rivers.^32^ This phenomenon was attributed to the distinct impacts of environmental factors, such as soil properties, temperature, rainfall patterns, and aerosol deposition, on the mobilization of DOC and DBC.^38^ Within the context of the studied rivers, the occurrence of sudden wildfire incidents likely intensified the correlation between DBC and DOC. Meanwhile, the strong coupling between atmospheric OC and BC during wildfires in this area (Figure S4) also indicated that riverine DOC and DBC were likely sourced from ash deposition. Studies have also found a coupling of DOC and certain water quality parameters, such as turbidity,^13,14^ suggesting that DOM likely originated from mobilized surface materials due to postfire runoff and erosion.^13^ Salinity has been widely documented to negatively correlate with aquatic DBC via affecting the physical and photochemical processes of DBC.^39,40^ Notably, there were no significant correlations between tested water quality parameters and DOC or DBC (Figures S5 and S6), indicating that their dynamics were decoupled from DOC or DBC. The decoupling indicated that these parameters might be less influenced by ash deposition, and riverine DOC and DBC deposited immediately after wildfires were less affected by water chemistry.

The BPCA distributions of DBC in Chongqing Jialing and the Yangtze River are shown in Figure 2b. Through nitric oxidation, the formation of highly substituted BPCAs (i.e., B6CA and B5CA) occurs when aromatic rings are surrounded by multiple aromatic rings, while poorly substituted BPCAs (i.e., B3CA) form when aromatic rings are surrounded by only a few aromatic rings.^26,41^ In comparison to B3CA and B4CA, B5CA and B6CA serve as indicators of DBC with a more pronounced aromatic structure and increased condensation.^28,42^ In the studied rivers, the predominant BPCAs were B5CAs (19.0, 71.7%) and B6CA (8.0, 64.1%), followed by B3CA (4.9, 37.9%) and B4CA (4.8–21.5%). The degree of condensed aromaticity of DBC was further estimated using the ratio of (B6CA + B5CA)/(B4CA + B3CA) (Table 1). The BPCA ratio is associated with pyrogenic source material and prevailing environmental conditions.^43^ For example, a higher BPCA ratio, indicating more condensed aromatic DBC, is linked to DBC originating from watersheds affected by wildfires,^43^ whereas DBC originated from urban dust and fossil fuel combustion exhibits a lower ratio.^11,25^ Furthermore, environmental factors such as prolonged solar radiation exposure^28^ and elevated salinity levels^44,45^ are linked to relatively low BPCA ratios. In the studied rivers, the BPCA ratios ranged from 0.83 to 7.54, surpassing the reported values in rivers of the Bohai Rim region (0.40–0.55),^45,46^ and surface waters of Alpine, Antarctic, and Arctic regions (0.02–0.77).^25^ Thus, it is reasonable to infer that the abrupt wildfires played a role in introducing riverine DBC with high condensed aromaticity. Given that the samples were collected shortly after the wildfires, the pronounced condensed aromaticity of DBC appeared to be minimally influenced by the water conditions or potential photodegradation from solar radiation. Notably, at locations L1, L3, L5, and L6 with high DBC concentrations and DBC/DOC, there was a corresponding high degree of polycondensation of DBC (Table 1).

### General Molecular Characterization of DOM Using FT-ICR-MS

Using FT-ICR-MS, a total of 10,280 unique molecular formulas were identified across all DOM samples, of which 19.7% were common/shared, while 41.7% were unique and distinct from each other. Generally, each DOM sample contained a range of 4675–5262 unique molecular formulas. The CHO compounds were the most abundant species of DOM, constituting 2053–2575 (39.7–54.8%) of the total assigned molecular formulas, and 56.0–82.0% of the intensity-averaged abundance. The CHO compounds contain typical products resulting from incomplete combustion,^48^ have been widely detected in the ESI negative mode and identified in aquatic DOM.^49,50^ Notably, DOM samples from L1, L3, L5, and L6 with higher DOC concentrations contained more molecular formulas (5007–5262 vs 4675–4714). However, the CHO compounds from locations L1, L3, L5, and L6 contained lower number of formulas (2052–2381, 39.7–47.0%) compared to locations L2, L4, and L7 (2528–2575, 54.1–54.8%). As a result, wildfires potentially enhanced the molecular complexity and diversity of DOM but reduced the diversity and abundance of CHO compounds in the studied rivers.^49,51^ Biomass combustion that introduces oxygen to a molecule can reduce its volatility and hydrophobicity, subsequently increasing its water solubility.^52^ The high degree of oxidation was indicative of the presence of acidic functional groups, such as hydroxyl and carboxylic acids.^22^ The majority of detected CHO compounds were heavily oxidized (O ≥ 10), constituting 56.3–66.9% of the total number of CHO formulas. However, locations L1, L3, L5, and L6 contained more low-oxygen-containing CHO species (O < 10) (Figure 4), indicating that the incomplete combustion of biomass caused by limited oxygen availability resulted in the formation of low-oxygen-containing CHO compounds.

**Figure 3:**
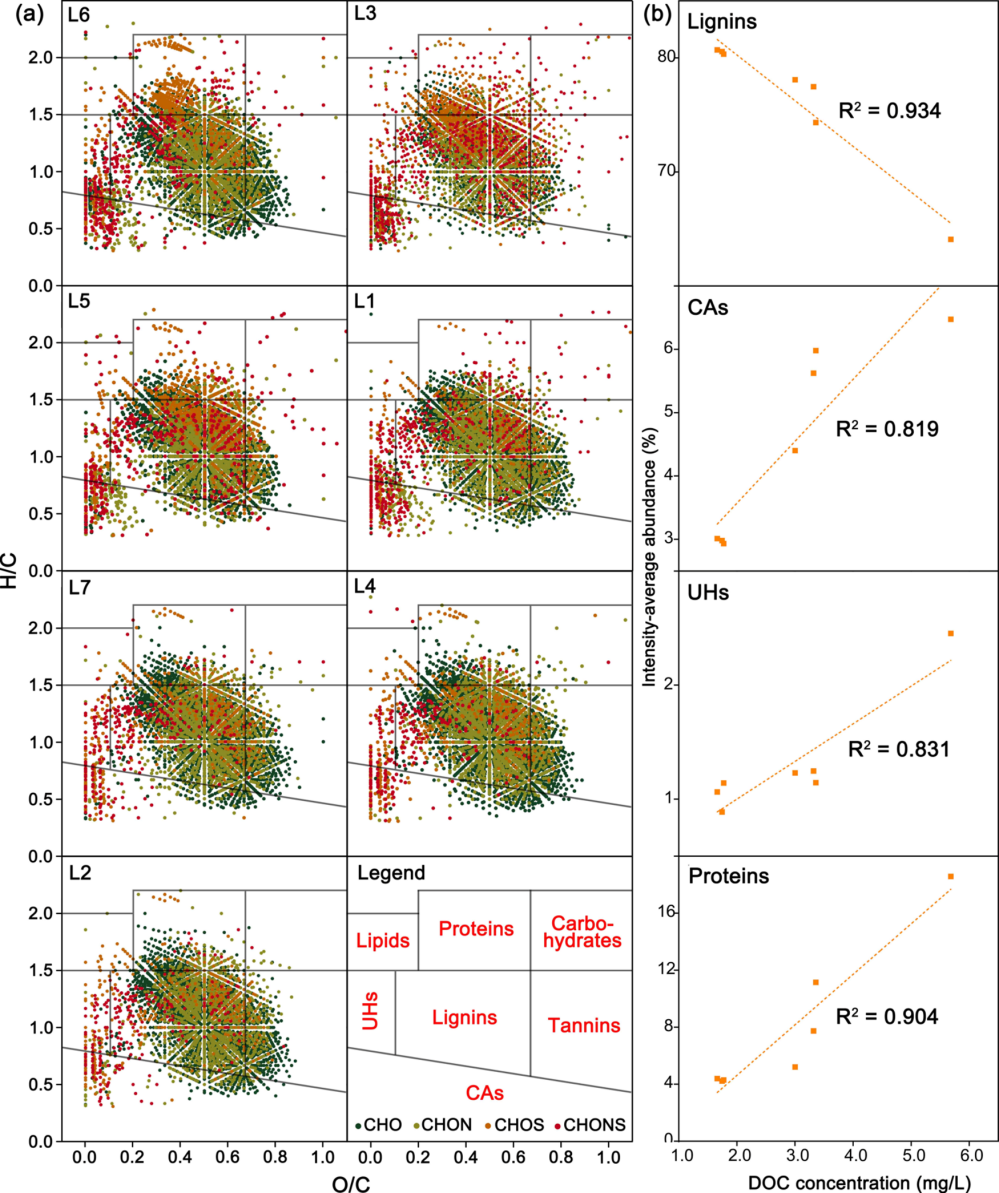
(a) van Krevelen diagrams of DOM from different locations. (b) Correlations between DOC concentration and intensity-averaged abundance of lignins, CAs, UHs, and proteins.

As shown in the van Krevelen diagram (Figure 3a), the dominant composition among DOM molecules was lignins, followed by tannins, protein-like compounds, CAs, UHs, carbohydrates, and lipids. Notably, in locations highly impacted by wildfires (L1, L3, L5, and L6), there were lower abundances of lignins (58.3–64.5 vs 66.9–67.5%) and tannins (8.9–12.4 vs 12.6–13.4%), yet higher abundances of protein-like compounds (8.8–13.4 vs 7.9–8.2%), CAs (11.2–11.6 vs 8.6–8.8%), and UHs (2.2–5.1 vs 1.8–2.2%) compared to L2, L4, and L7. Additionally, the intensity-averaged abundances of protein-like compounds, CAs, and UHs exhibited a strong positive correlation with the DOC concentration of DOM (*R*^2^ = 0.904, 0.831, and 819, respectively), whereas the abundance of lignins was negatively correlated with the DOC concentration (*R*^2^ = 0.934) (Figure 3b). It revealed a strong association between wildfires and the increase in protein-like compounds, CAs, and UHs, along with a decrease in lignins. Chen et al. also reported that the identified formulas in DOM from burned materials contained a higher proportion of proteins (7.26–8.14 vs 0.59–5.23%) and CAs (20.4–27.1 vs 3.04–4.67%) compared to unburned material.^49^ During wildfires, the conversion of plant-derived lignin/phenol to (poly)aromatic carbon accompanied by the loss of side chains was extensively documented, and as a result, there was an increase in aromaticity and a decrease in molecular weight.^49,51^ However, in this study, the aromaticity ((AI_mod_)_wa_) and double-bond equivalence (DBE_wa_) were negatively correlated with the DOC (Table S5). It was attributed to the production of low-aromaticity protein-like compounds, as evidenced by negative correlations between (AI_mod_)_wa_ and DBE_wa_ of protein-like compounds and DOC (Table S5). Notably, there was a strong negative correlation between DOC and the redox potential of DOM molecules (NOSC_wa_) (Table S5), indicating that DOM with a high DOC was more reductive. The decoupling of DOC and ORP of water (Figure S5) indicated that other factors may affect the redox state of the aquatic environment, independent of the DOM potentially derived from ash deposition.

### Nitrogen-Containing DOM Release under Wildfires Impacts

The CHON compounds were the second most frequently detected species, constituting 1484–1776 formulas, accounting for 31.7–35.5% of the total formulas (Table S6). A higher number of CHON formulas were detected in locations L1, L3, L5, and L6 relative to locations L2, L4, and L7 (1678–1776 vs 1484–1523). Chen et al. also reported that DOM from burned materials contained a higher percentage of CHON compounds compared to unburned materials (33.5–45.3% versus 5.56–14.1%).^49^ It was related to the creation of heterocyclic nitrogen compounds during the wildfires, leading to the preferential fixation of nitrogen over carbon.^53^ As reported, wildfires, particularly under flaming conditions, are known to release CHON species like nitrogen oxides (NOx) and nitrous acid (HONO).^54,55^ These short-lived compounds may subsequently undergo transformations into longer-lived compounds, such as peroxyacetyl nitrate (PAN), nitric acid (HNO_3_) organic nitrates, or other organic nitrogen-containing compounds.^54^ The CHON compounds were classified into 9 subgroups according to their N and O numbers, including N_1_O_*x*_, N_2_O_*x*_, N_3_O_*x*_, and N_4_O_*x*_. According to previous studies, an amine or nitro group typically has an O/N ratio lower than 3, while the high O/N ratios (≥3) of these formulas allow an assignment of one nitro (–NO_2_) or nitrooxy (–ONO_2_) group, and PAN exhibits an O/N ratio of 5 or higher.^56,57^ In this study, it was found that 81.0–94.0% of the CHON compounds had an O/N ratio of ≥ 3, and 71.4–86.1% had an O/N ratio of ≥ 5. Hence, it can be inferred that most of CHON compounds were likely PAN compounds, exhibiting quite high polarity and water solubility.^57^ Notably, fewer formulas with O/N ≥ 3 were found for L1, L3, L5, and L6 compared to L2, L4, and L7 (81.0–89.4 vs 92.6–94.0%). The difference was attributed to the N_1_O_*x*_ species, implying that wildfires contributed to the formation of more reduced N_1_O_*x*_ species. However, the abundances of both O/N < 3 and O/N ≥ 3 of N_2_O_x_ species were higher at locations highly affected by wildfires. Moreover, the CHON compounds at these locations contained more N_3_O_*x*_ and N_4_O_*x*_ species, almost all of which had O/N ratios of less than 3. Therefore, wildfires were strongly correlated with the generation of reduced nitrogen compounds, particularly those containing a higher number of nitrogen atoms. As reported, these reduced nitrogen compounds might be mainly associated with alkyl amino and alkyl nitrile as well as heterocyclic aromatic rings with a single N atom.^58,59^ Notably, the relative abundances of condensed aromatics within the CHON compounds were higher in locations L1, L3, L5, and L6 in contrast to L2, L4, and L7 (13.2–16.9 vs 10.0–11.6%). The elevated proportion of CAs in these locations was linked to the reduced abundance of oxygen-rich tannins and/or lignins, while no notable alterations were observed in the proportions of UHs, proteins, and carbohydrates.

**Figure 4:**
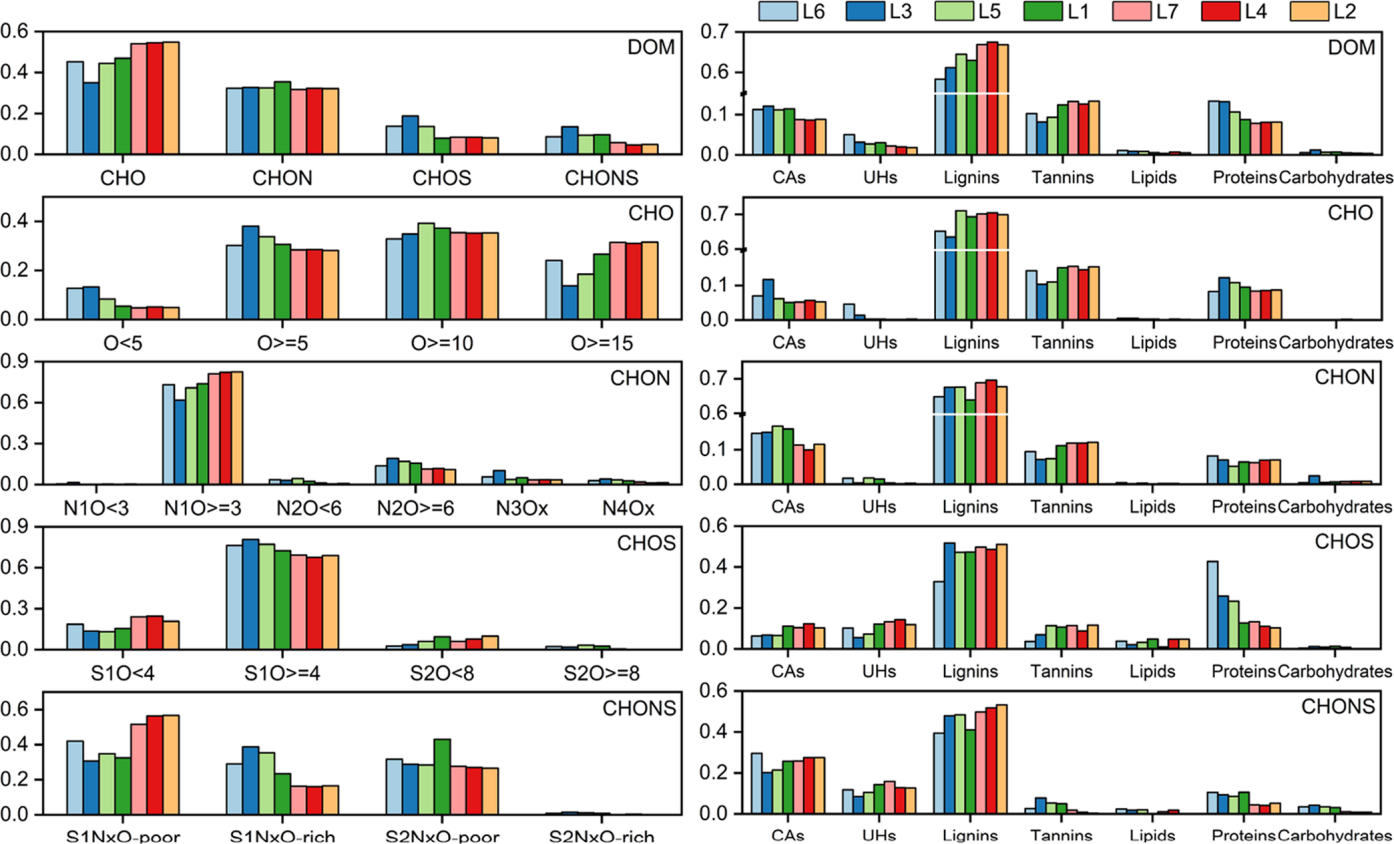
(Left) Formula number proportion of elemental composition-based subgroups for respective DOM, CHO, CHON, CHOS, and CHONS compounds; (Right) Formula number proportion of seven compound classifications for respective DOM, CHO, CHON, CHOS, and CHONS compounds.

CAs can be divided into CHO, CHON, CHOS, and CHONS species based on the presence of heteroatoms (Figures S10 and S11). Only 20.9–35.9% of the formulas of CAs were characterized as CHO species, while the other 64.1–79.1% contained CHO and one or more heteroatoms such as N and S. The CHON and CHOS species of CAs are also known as dissolved black nitrogen (DBN) and dissolved black sulfur (DBS), originating from the incorporating of organic nitrogen and sulfur into DBC during biomass burning.^12,60^ Similar to CHO species, the DBS formula numbers exhibited no significant discrepancy between locations impacted and unimpacted by wildfires (Figure S10). Remarkably, DBN constituted the highest abundance, accounting for 37.3–49.7% (152–286 formulas) of the total CAs. More DBN formulas (227–286) were detected in locations highly affected by wildfires compared to other locations (152–175). A study also revealed that the levels of DBN within DOM from burned biomass were 5.8 to 21.2 times higher than those found in unburned biomass,^49^ highlighting the significant role of wildfires in the formation of DBN. Additionally, relatively high formulas (104–142 vs 60–70) of the CHONS species were also detected in locations highly affected by wildfires. The CHONS species of CAs also partially explained the increased CHONS compounds within the DOM under the impacts of wildfires (Figure 4).

### Sulfur-Containing DOM Release under Wildfires Impacts

The CHOS (379–979 formulas) and CHONS compounds (218–703 formulas) accounted for 7.9–18.8% and 4.6–13.5% of the total identified compounds in studied rivers, respectively (Figure 4). Notably, locations highly affected by wildfires (L3, L5, and L6) exhibited a greater number of CHOS formulas, ranging from 704 to 979, compared to other locations with 379–399 formulas. The intensity-averaged abundance of CHOS was also diverse among the tested samples, with higher percentages of CHOS compounds in locations L1, L3, L5, and L6 compared to other locations (4.1–4.6 vs 8.9–18.1%). Similarly, locations L1, L3, L5, and L6 contained more CHONS species relative to locations L2, L4, and L7 (456–703 vs 218–271, 8.7–13.5 vs 4.6–5.8%). Sulfur that can be sourced from both the soil and the atmosphere in the form of SO_2_ and sulfate is a vital nutrient for plants as a part of biomass such as disulfide bonds in plant proteins and a range of other sulfur-containing (as well as oxygen- and nitrogen-containing) compounds.^61^ Although there have been limited studies on the specific contribution of wildfires to the release of sulfur-containing compounds into rivers, existing research suggested that biomass burning plays an important role in the emission of sulfur-containing compounds to the atmosphere.^48,57,61^ In this study, due to atmospheric deposition, wildfires might contribute to the input of abundant sulfur-containing compounds into the rivers.

The identified CHOS formulas were divided into four subgroups based on their S and O numbers (Figure 4). The O-rich CHOS fraction (O/S ≥ 4) typically contains organic sulfates (−OSO_3_H) or sulfonates (−SO_3_), whereas when O/S < 4, it suggests the presence of less oxidized sulfur functional groups such as sulfones (−SO_2_−), sulfonic acid (−SO_3_H), sulfinic acid (−SO_2_H), or sulfides.^48^ 67.7–80.6% of CHOS formulas was identified as O-rich species, with intensity-averaged abundance of 57.3–91.2%, indicating that most of the CHOS compounds detected by negative ESI sources likely contained sulfate (−OSO_3_H) or sulfonate (−SO_3_) groups. Based on N, S, and O atoms, CHONS compounds were classified into O-poor ((4S + 3N)/O < 1) and O-rich ((4S + 3N)/O ≥ 1) compounds. The O-rich CHONS compounds are likely nitrooxy–organosulfates containing nitrate (–ONO_2_) groups, while O-poor species might contain reduced sulfur (e.g., sulfones or aromatic sulfur) or reduced nitrogen functional groups (e.g., amine or heterocyclic aromatics) due to their insufficient oxygen content in the molecular formulas.^57^ As the majority of CHONS compounds were O-poor compounds that accounted for 59.6–83.5% of total formulas, these compounds mostly contained reduced nitrogen and sulfur functional groups. Ditto et al. also indicated that the CHONS compounds emitted from a forest fire contained abundant aromatic nitrogen, amine, and imine, as well as sulfide, aromatic sulfur, and sulfone functional groups.^61^

Although there were more both O-poor and O-rich sulfur-containing formulas observed in locations affected by wildfires, it is important to note that wildfires potentially increased the relative abundance of both the O-rich CHOS and CHONS compounds, as indicated by the more abundant of the O-rich species, especially those containing one S atom, in locations L1, L3, L5, and L6 compared to L2, L4, and L6 (Figure 4). In addition, for both CHOS and CHONS compounds, the relatively high O/C_wa_ and H/C_wa_ but low DBE/C_wa_ and (AI_mod_)_wa_ were found in locations highly affected by wildfires (Table S6), indicating the formation of oxidative, aliphatic, saturated, and low-aromatic S-containing compounds under the impacts of wildfires. In locations L1, L3, L5, and L6, there was an increased presence of sulfur-containing lignins and protein-like compound formulas compared to other locations. In terms of relative abundance, higher percentages of protein-like substances were detected in these wildfire-impacted areas (Figure 3). In particular, at location L6, the abundance of protein-like CHOS compound formulas and their intensity-averaged abundance were notably high, reaching 44.1% and 80.7%, respectively.

## Environmental Implications

The globally increasing extreme high temperatures in recent decades have led to a rise in wildfires, posing a significant threat to global ecosystems and impacting ecological security. Wildfires typically transport pyrogenic organic matter and nutrients to rivers through interactions with the hydrological cycle.^13^ Our study highlighted that atmosphere ash deposition was likely responsible for the high levels of DOC and DBC in locations close to the wildfire areas, but wind direction on the sampling day also played a role. The coupling of DOC and DBC, along with the decoupling of DOC and other water quality parameters, indicated that ash deposition might not significantly affect water chemistry but might contribute to aquatic DOM/DBC dynamics. Besides, DOM/DBC sourced from abrupt wildfires was not significantly affected by the original water chemistry in the short term. Based on the molecular composition analysis, the majority of DBC was DBN which mainly accounted for the increase in N-containing DOM. Moreover, sulfur-containing substances in plants were integrated into the pyrogenic OM during wildfires, contributing to the generation of abundant recalcitrant DOS.

Such pyrogenic DBN and DOS may be more recalcitrant to photodegradation and biotransformation than the bulk DOM and thus accumulate in aquatic systems.^62,63^ These substances may further influence various aspects of water chemistry, such as pH^64,65^ and redox potential,^66^ as well as the biogeochemical cycling of trace metals.^67^ For instance, a strong association was observed between DBN/DOS abundance and the reductive activity of DOM molecules. It suggested that these pyrogenic DOM have the potential to alter the water redox potential and element cycling, despite no relationships being observed in the short term. In addition, the high photoactivity of pyrogenic DOM in the DOM pool may contribute to contaminant photodegradation via photosensitivity processes.^68,69^ The appearance of riverine pyrogenic DOM may modify microbial loop dynamics, shaping the structure and function of riverine microbial communities, including processes related to nitrogen and sulfur cycling.^70−72^ Therefore, the wildfire-derived pyrogenic DOM may potentially impact water quality and environmental biogeochemistry in various ways. Further research is needed to understand their specific effects and contributions to ecosystem processes in the long term. A comprehensive understanding of the fate and transformation, including aspects such as photoreactivity and bioavailability, of wildfires-derived pyrogenic DOM is crucial for a more thorough comprehension of its environmental implications.

## Supporting Information Available

Analysis of FT-ICR-MS data (Text S1); Chongqing’s map and location on the map of China (Figure S1); pictures of several sampling locations (Figure S2); amount of precipitation (mm) in Chongqing before and after wildfire events (Figure S3); daily average atmospheric OC and BC concentrations in Chongqing before and after wildfire events (Figure S4); correlation analysis of tested water quality parameters and DOC (Figure S5); correlation analysis of tested water quality parameters and DBC (Figure S6); full spectra of (−) ESI FT-ICR-MS of PPL-DOMs at different locations (Figure S7); intensity-averaged proportion of elemental composition-based subgroups for respective DOM, CHO, CHON, CHOS, and CHONS compounds, and intensity-averaged proportion of seven compound classifications for respective DOM, CHO, CHON, CHOS, and CHONS compounds (Figure S8); correction between DOC concentration of DOM and intensity-averaged abundance of tannins, carbohydrates, and lipids (Figure S9); formula number of elemental composition-based subgroups for respective DOM, CAs, UHs, lignins, tannins, lipids, proteins, and carbohydrates (Figure S10); formula number proportion of elemental composition-based subgroups for respective DOM, CAs, UHs, lignins, tannins, lipids, proteins, and carbohydrates (Figure S11); intensity-averaged proportion of elemental composition-based subgroups for respective DOM, CAs, UHs, lignins, tannins, lipids, proteins, and carbohydrates (Figure S12); information about sampling locations (Table S1); mobile phase mixing gradients (Table S2); calibration curves of BPCAs target compounds (Table S3); molecular parameters of DOM with different molecular compositions (Table S4); correlations between DOC and molecular parameters of DOM with different molecular compositions (Table S5); molecular parameters of DOM with different elemental compositions (Table S6); and correlations between DOC and molecular parameters of DOM with different elemental compositions (Table S7) (PDF)
