# Supplementary material for: Appearance of Recalcitrant Dissolved Black Carbon and Dissolved Organic Sulfur in River Waters Following Wildfire Events
Source: Environ Sci Technol. 2024 Apr 10;58(16):7165–75. doi: 10.1021/acs.est.4c00492 (PMC11044583; doi:10.1021/acs.est.4c00492)
Supplement: Supplementary file 1 — es4c00492_si_001.pdf [file es4c00492_si_001.pdf]

Supplementary materials for:

Appearance of Recalcitrant Dissolved Black Carbon and Dissolved Organic Sulfur in River Waters Following Wildfire Events

Yanghui Xu [a, b, #] & Xintu Wang [a, c, #], Qin Ou [a, b], Zhongbo Zhou [d *], Jan Peter van der Hoek [b, e], Gang Liu [a, b, f *]

[a] *Key Laboratory of Drinking Water Science and Technology, Research Centre for Eco-Environmental Sciences, Chinese Academy of Sciences, Beijing, 100085, P. R. China*

[b] *Section of Sanitary Engineering, Department of Water Management, Faculty of Civil Engineering and Geosciences, Delft University of Technology, Stevinweg 1, 2628 CN Delft, the Netherlands*

[c] *College of Environmental Science and Engineering, Guilin University of Technology, Guangxi, 541004, China*

[d] *College of Resources and Environment, Southwest University, Chongqing 400715, China*

[e] *Waternet, Department Research & Innovation, P.O. Box 94370, 1090 GJ Amsterdam, the Netherlands*

[f] *University of Chinese Academy of Sciences, Beijing, China*

[#] *Yanghui Xu and Xintu Wang contributed equally to this manuscript*

*Corresponding authors：

Prof. Dr. Gang Liu,

Research Center for Eco-Environmental Sciences,

Chinese Academy of Sciences,

Beijing, China

email: gliu@rcees.ac.cn

Tel: 008617600879707

The supplementary material includes 1 text, 12 figures, and 7 tables in 15 Pages.

Text S1. Analysis of FT-ICR-MS data

Double bond equivalents (DBE) must be a whole number. The calculation of DBE is as follows:[1, 2]

$$DBE = 1 + C - 0.5H + 0.5N \quad (1)$$

The normal oxidation state of carbon (NOSC) was used to reflect the redox potential of a given formula. The positive values mean the compounds at the oxidized state, the negative values mean that at the reduced state, and zero means that it is in this neutral state.[3] The nominal oxidation state of carbon (NOSC) was calculated based on the equation:[4]

$$NOSC = 4 - 4C + H - 2O - 3N - 2S \quad (2)$$

Based on the assigned formulae of each DOM molecule, the intensity-weighted average (wa) parameters such as double-bond equivalence (DBEwa), molecular weight (MWwa), H/Cwa, O/Cwa were calculated based on the following equations:[2-4]

$$DBE_{wa}=\sum(DBE_n \times M_n) \quad (3)$$

$$Mw_{wa}=\sum(Mw_n \times M_n) \quad (4)$$

$$O/C_{wa}=\sum(O/C_n \times M_n) \quad (5)$$

$$H/C_{wa}=\sum(H/C_n \times M_n) \quad (6)$$

$$(AI_{mod})_{wa}=\sum((AI_{mod})_n \times M_n) \quad (7)$$

$$NOSC_{wa}=\sum(NOSC_n \times M_n) \quad (8)$$

$$DBC\text{-}C_{wa}\ (\%) = \sum(DBC\text{-}C_n \times M_n)/ \sum(DOM\text{-}C_n \times M_n) \quad (9)$$

Where wa means an intensity-averaged calculation; n means list number of each assigned molecular formula; M is the relative intensity of each formula.

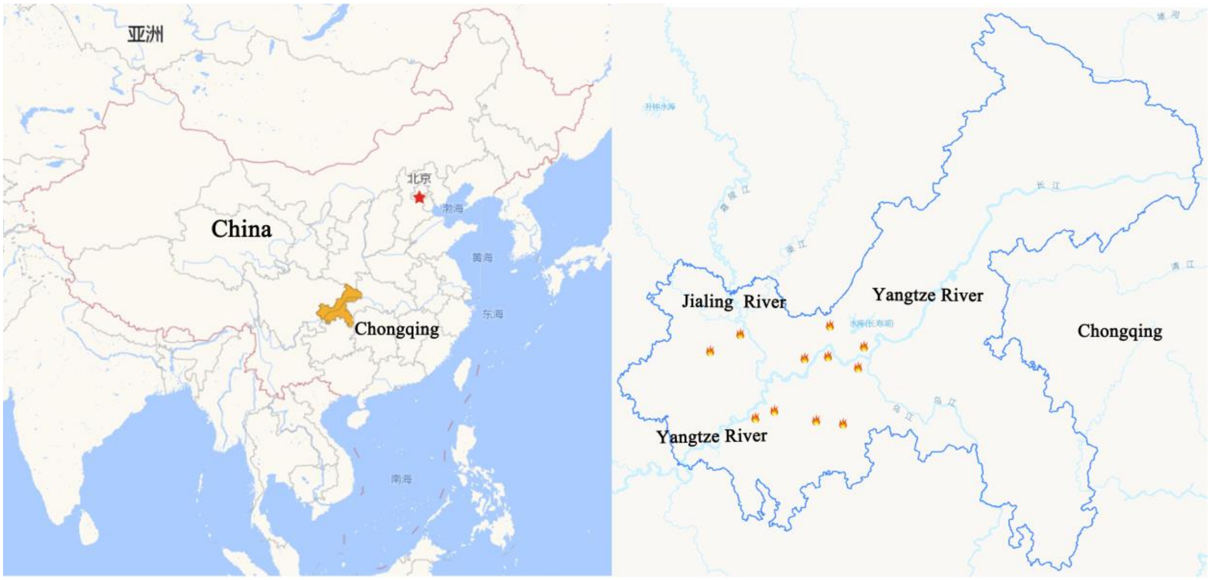

Figure S1. Chongqing's map and location on the map of China.

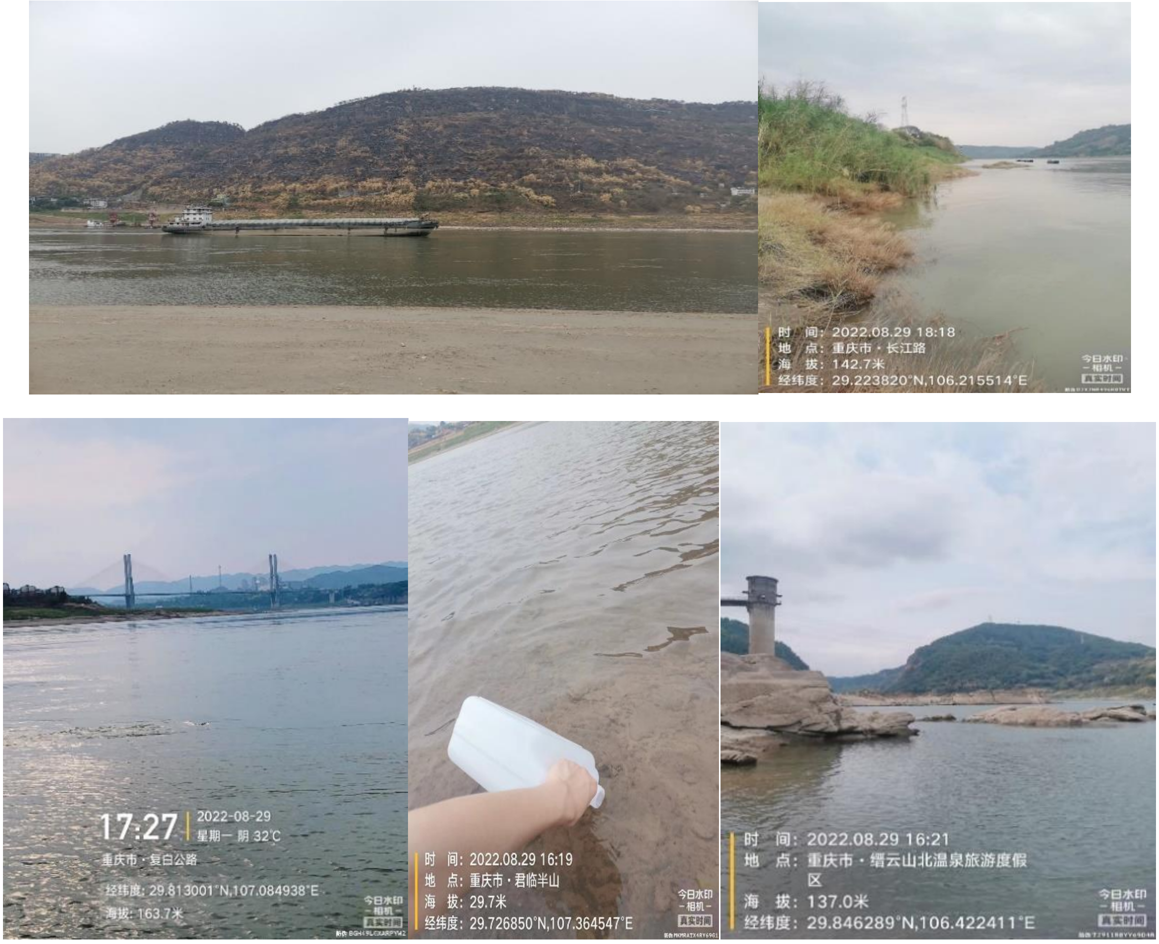

Figure S2. Pictures of several sampling locations.

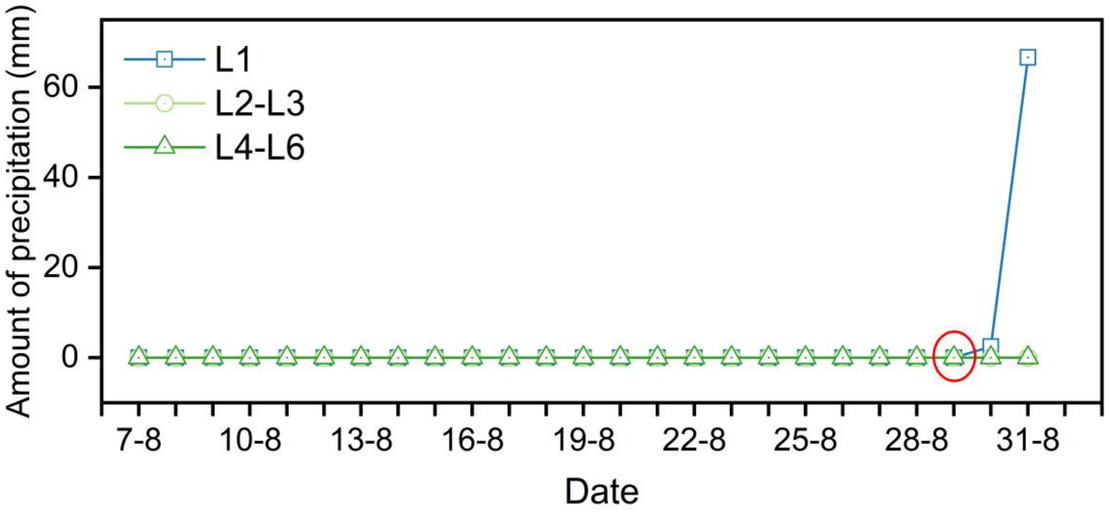

Figure S3. The amount of precipitation (mm) in Chongqing before and after wildfire events. Data were obtained from the weather records in China (https://www.tianqi24.com/historycity/).

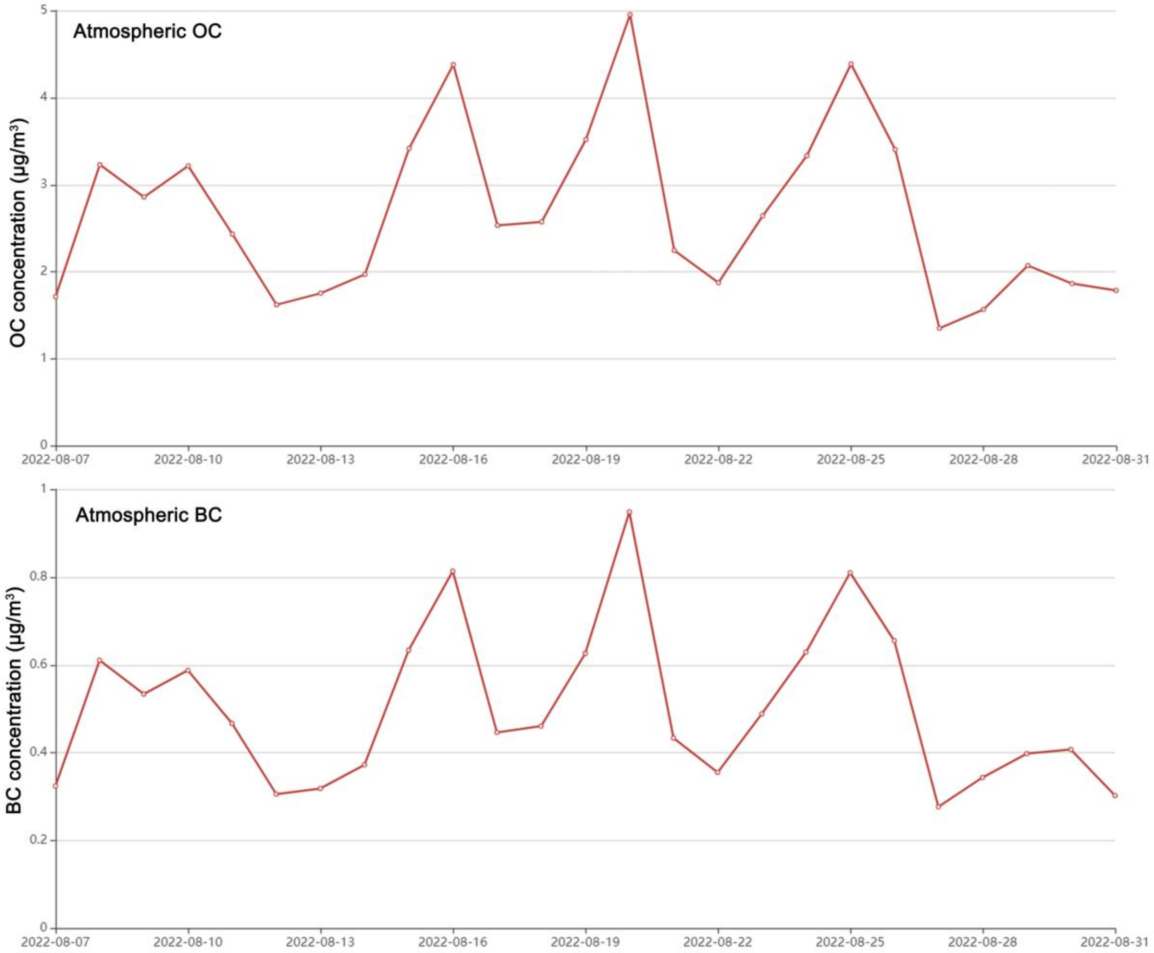

Figure S4. Daily average atmospheric OC and BC concentrations in Chongqing before and after wildfire events. Data obtained from the Tracking Air Pollution in China dataset (TAP, http://tapdata.org.cn/).

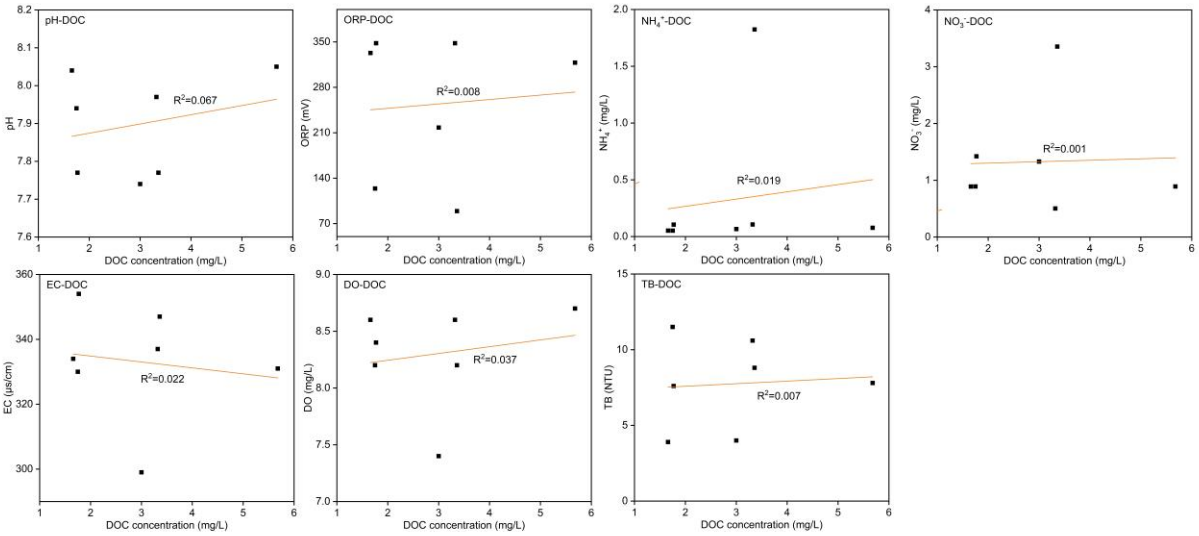

Figure S5. The correlation analysis of tested water quality parameters and DOC

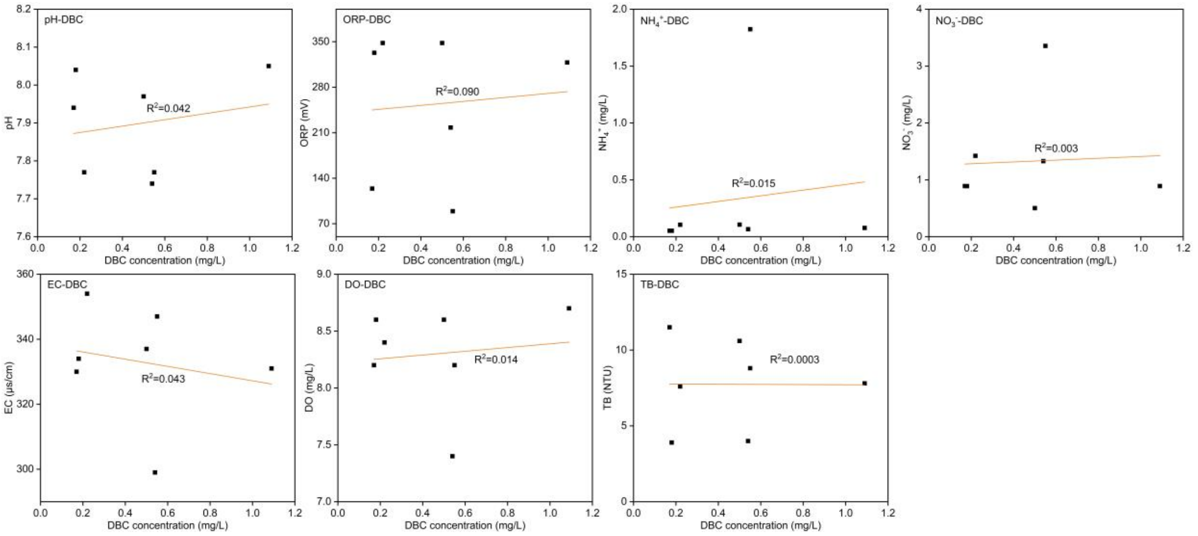

Figure S6. The correlation analysis of tested water quality parameters and DBC.

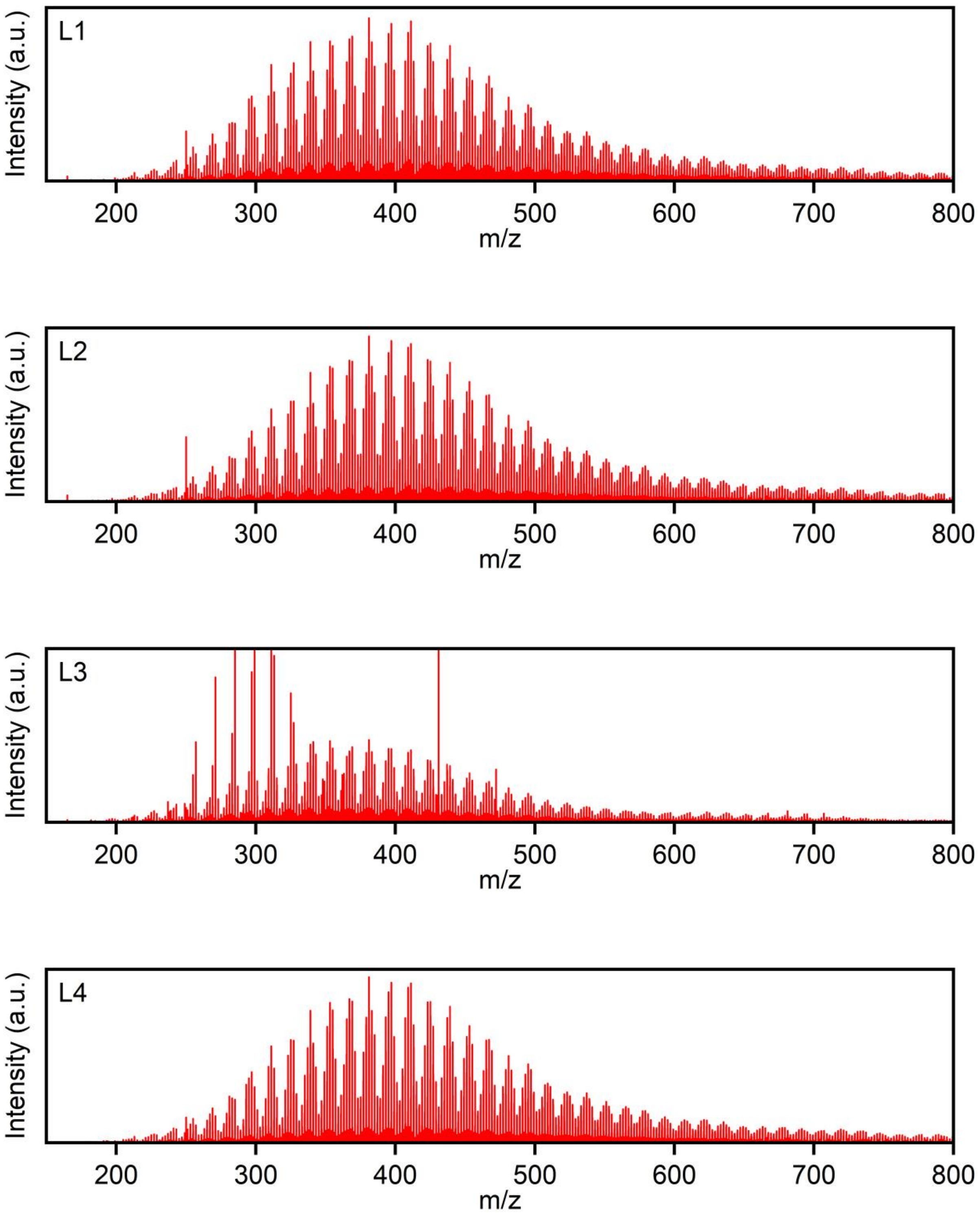
L1
L2
L3
L4
Intensity (a.u.)
m/z
200
300
400
500
600
700
800

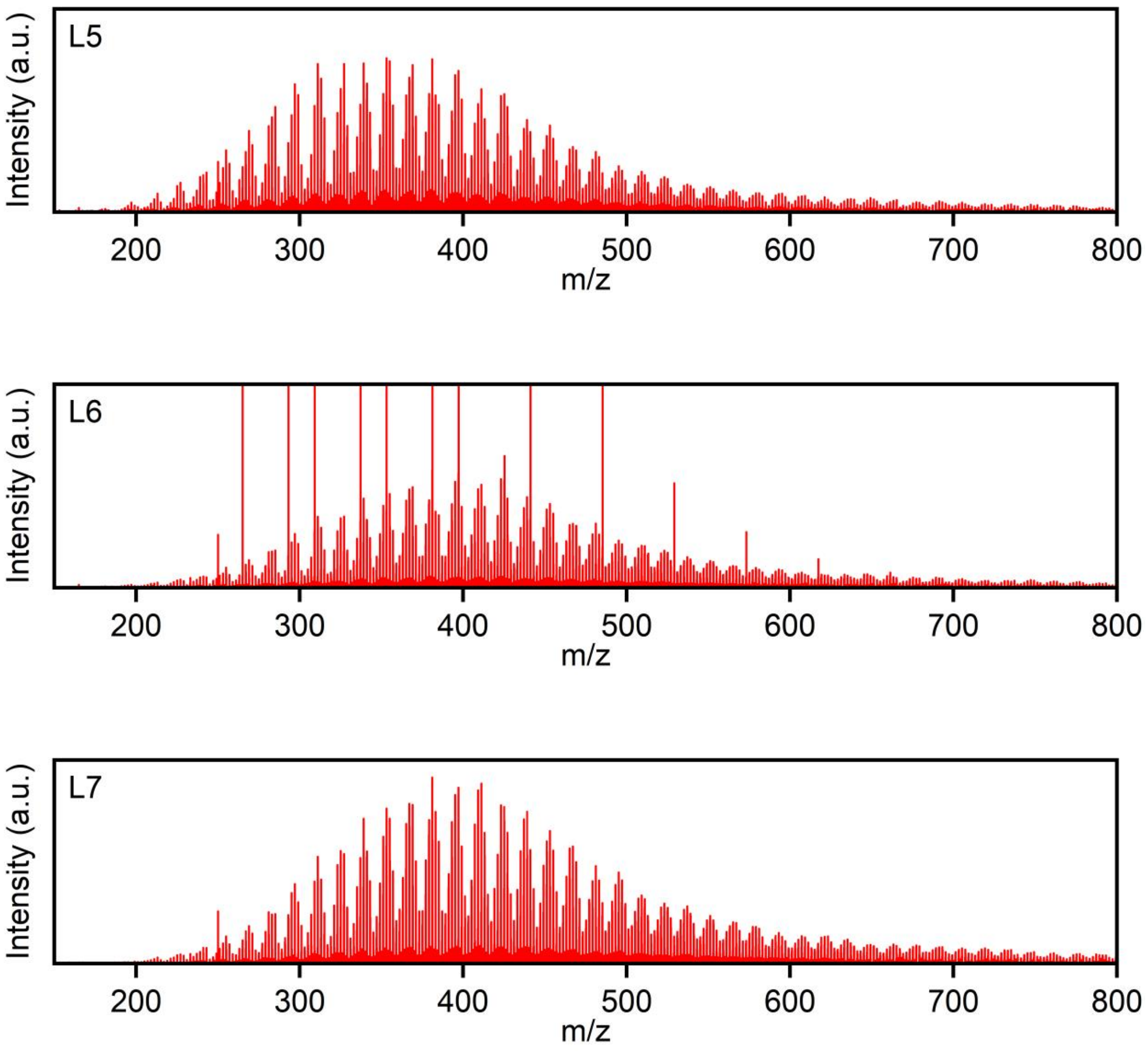

Figure S7. Full spectra of (–) ESI FT-ICR-MS of PPL-DOMs at different locations.

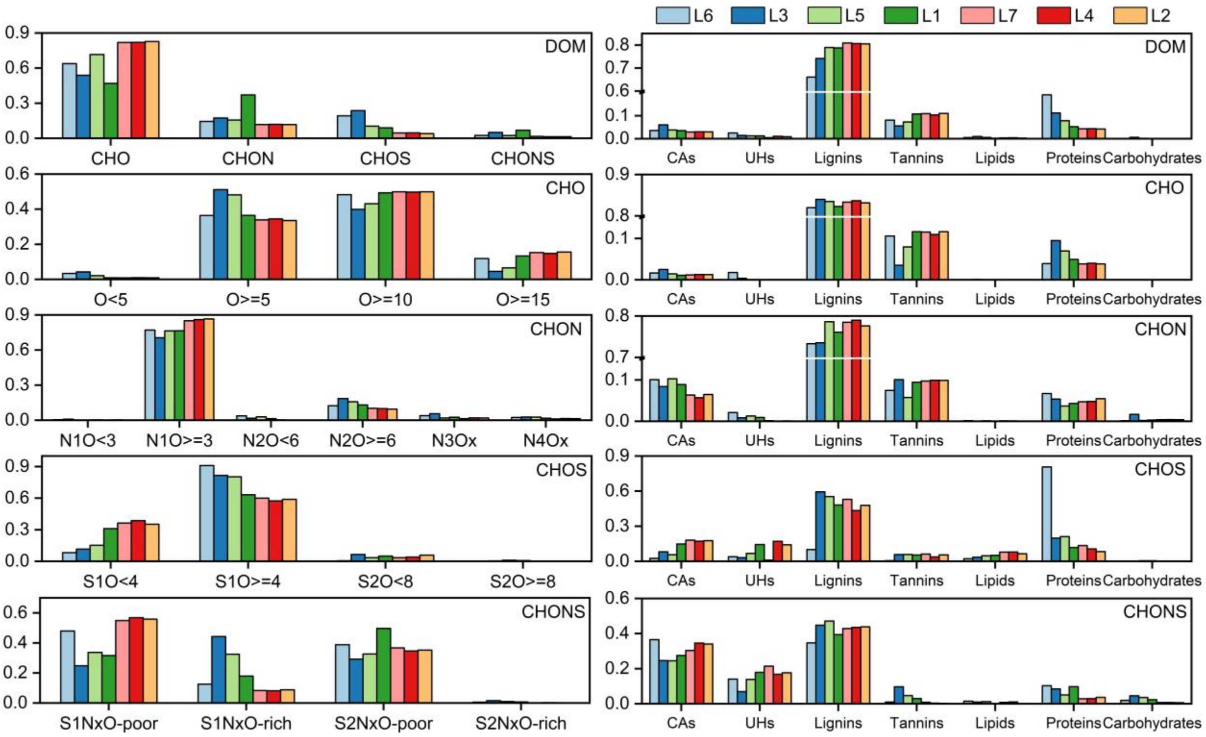

Figure S8. (Left) The intensity-averaged proportion of elemental composition-based subgroups for respective DOM, CHO, CHON, CHOS, and CHONS compounds; (Right) The intensity-averaged proportion of seven compound classifications for respective DOM, CHO, CHON, CHOS, and CHONS compounds.

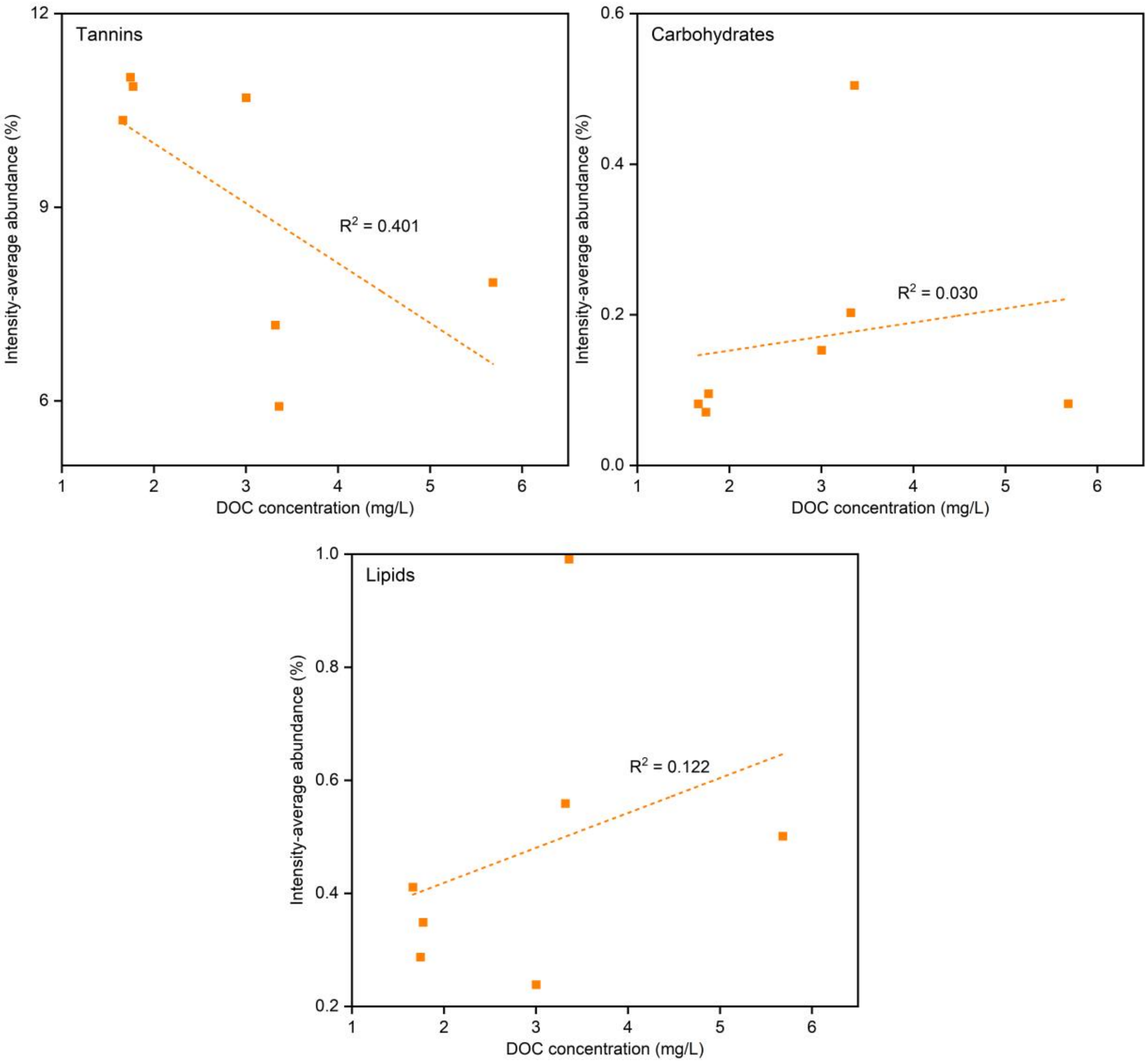

Figure S9. The correction between DOC concentration of DOM and intensity-averaged abundance of tannins, carbohydrates and lipids.

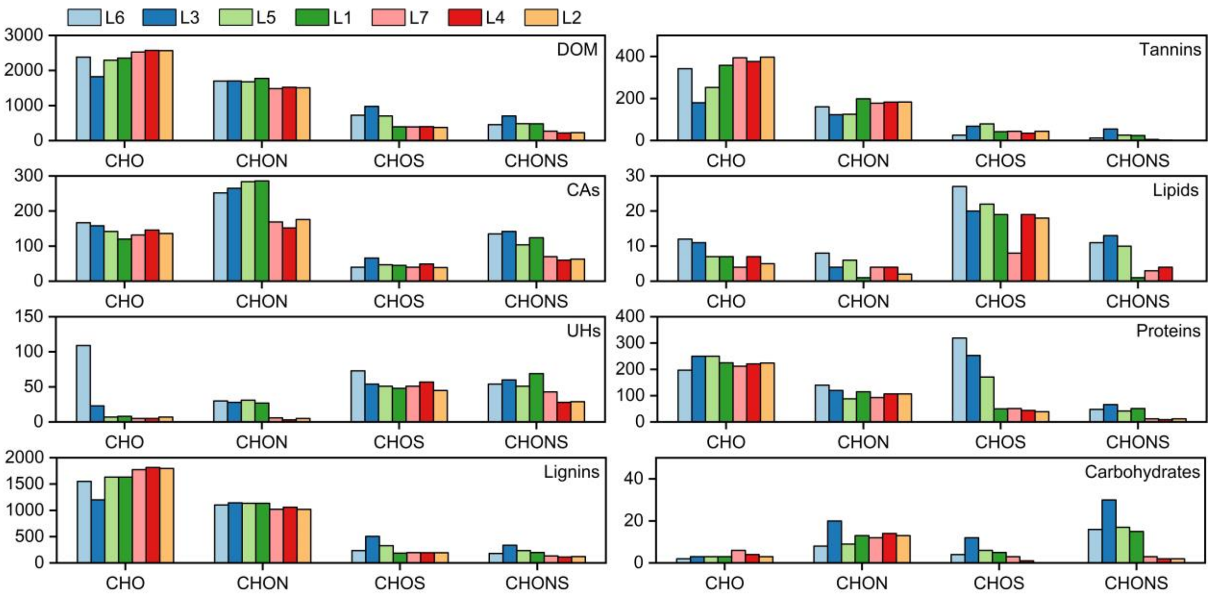

Figure S10. Formula number of elemental composition-based subgroups for respective DOM, CAs, UHs, lignins, tannins, lipids, proteins, and carbohydrates

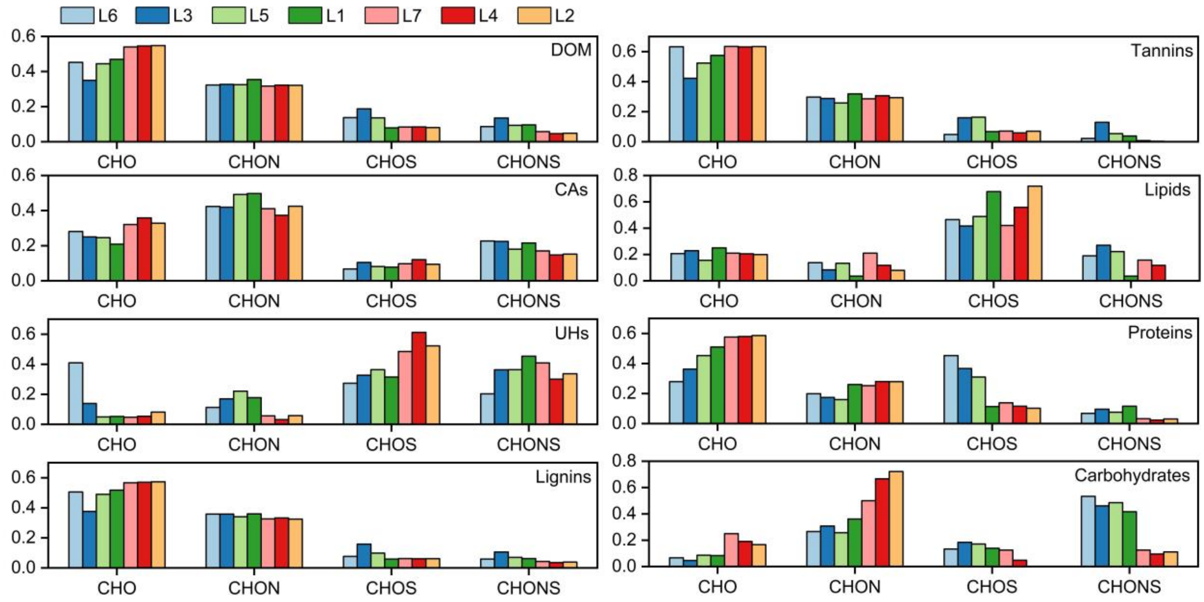

Figure S11. Formula number proportion of elemental composition-based subgroups for respective DOM, CAs, UHs, lignins, tannins, lipids, proteins, and carbohydrates

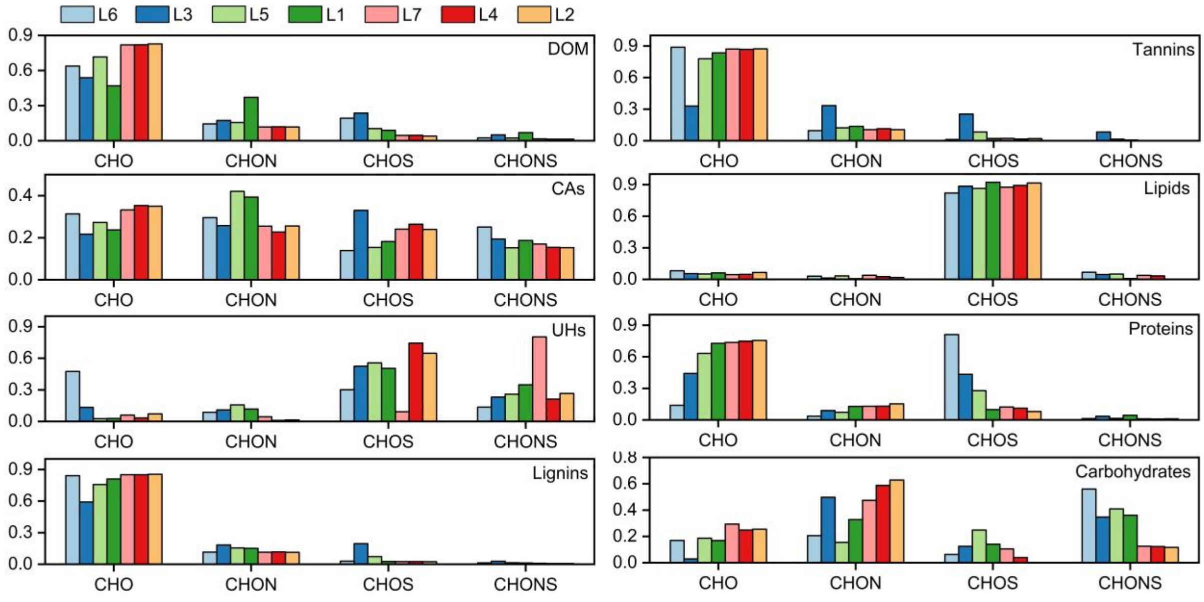

Figure S12. Intensity-averaged proportion of elemental composition-based subgroups for respective DOM, CAs, UHs, lignins, tannins, lipids, proteins, and carbohydrates

Table S1. Information about sampling locations.

Sampling location	N	E	Distance to the nearest wildfire site (km)	Fire severity of the nearest wildfire site[a]
L1	29.846	106.422	4.7	moderate
	29.821	106.445	4.8	
	29.836	106.438	5.2	
L2	29.225	106.215	21.2	low
	29.225	106.216	21.6	
L3	29.304	106.383	2.8	low
	29.304	106.383	3.0	
L4	29.813	107.083	6.9	low
L5	29.777	107.105	3.5	low
L6	29.755	107.099	2.5	low
L7	29.927	107.365	2.3	moderate
	29.716	107.384	1.1	
	29.719	107.404	1.8	

Note. a. the fire severity is referred to the study of Zhao et al.[5]

Table S2. Mobile phase mixing gradients.

Time (min)	Mobile phase A (vol.%)	Mobile phase B (vol.%)	Flow rate (mL/min)
0	90	10	0.5
8	80	20	0.5
18	80	20	0.5
18.01	20	80	0.5
28	20	80	0.5
28.01	90	10	0.5
35	90	10	0.5

Table S3. Calibration curves of BPCAs target compounds.

Target compounds	Retention time (min)	Calibration curve	Range (mg)	R^2
benzene-1,2,3,4,5,6-hexacarboxylic acid	3.399	y = 21.124x + 94.785	1-50	0.9969
benzene-1,2,3,4,5-pentacarboxylic acid	4.249	y = 41.166x + 2.8594	0.2-50	0.9991
benzene-1,2,4,5-tetracarboxylic acid	6.662	y = 31.19x + 15.805	0.2-50	0.9986
benzene-1,2,3,4-tetracarboxylic acid	7.764	y = 31.19x + 15.805	0.2-50	0.9986
benzene-1,2,3,5-tetracarboxylic acid	8.348	y = 31.19x + 15.805	0.2-50	0.9986
benzene-1,2,4-tricarboxylic acid	12.149	y = 44.971x + 11.028	0.2-50	0.9997
benzene-1,2,3-tricarboxylic acid	14.646	y = 22.141x + 3.2211	0.2-50	0.9999
benzene-1,3,5-tricarboxylic acid	21.013	y = 35.343x + 22.156	0.2-50	0.9996
biphenyl-2′2-dicarboxylic acid	29.408	–	–	–

Table S4. Molecular parameters of DOM with different molecular compositions.

DOM	Formulas	Mw_{wa}	O/C_{wa}	H/C_{wa}	N_{wa}	S_{wa}	DBE_{wa}	$(AI_{mod})_{wa}$	$NOSC_{wa}$
L6	5262	439.914	0.464	1.275	0.210	0.228	9.263	0.226	-0.292
L3	5171	412.657	0.445	1.205	0.286	0.283	9.420	0.280	-0.234
L5	5161	416.544	0.484	1.182	0.259	0.140	9.332	0.288	-0.159
L1	5007	452.213	0.498	1.150	0.261	0.082	10.309	0.301	-0.109
L7	4675	456.714	0.500	1.144	0.189	0.070	10.353	0.304	-0.108
L4	4714	454.941	0.499	1.143	0.180	0.066	10.338	0.305	-0.110
L2	4683	457.604	0.503	1.138	0.176	0.061	10.419	0.307	-0.098

CAs	Formulas	Mw_{wa}	O/C_{wa}	H/C_{wa}	N_{wa}	S_{wa}	DBE_{wa}	$(AI_{mod})_{wa}$	$NOSC_{wa}$
L6	580	471.453	0.253	0.607	1.331	0.511	21.371	0.744	0.091
L3	599	468.170	0.190	0.532	0.513	0.606	24.201	0.775	-0.013
L5	577	430.138	0.305	0.612	1.218	0.388	19.148	0.744	0.196
L1	575	462.567	0.299	0.606	1.222	0.479	19.864	0.737	0.168
L7	411	401.648	0.310	0.597	0.898	0.456	17.377	0.738	0.188
L4	407	400.213	0.324	0.596	0.743	0.449	17.137	0.729	0.190
L2	413	402.161	0.329	0.601	0.836	0.437	17.114	0.731	0.210

Tannins	Formulas	Mw_{wa}	O/C_{wa}	H/C_{wa}	N_{wa}	S_{wa}	DBE_{wa}	$(AI_{mod})_{wa}$	$NOSC_{wa}$
L6	460	461.638	0.705	0.980	0.104	0.015	10.818	0.330	0.450
L3	458	434.491	0.704	1.032	0.304	0.182	9.760	0.286	0.470
L5	405	421.908	0.701	1.006	0.158	0.101	9.699	0.315	0.440
L1	623	463.069	0.710	0.987	0.158	0.029	10.762	0.323	0.465
L7	621	469.938	0.710	0.980	0.112	0.024	10.978	0.326	0.462
L4	596	468.930	0.708	0.975	0.120	0.017	11.012	0.331	0.464
L2	626	474.257	0.710	0.973	0.110	0.021	11.152	0.331	0.468

Lignins	Formulas	Mw_{wa}	O/C_{wa}	H/C_{wa}	N_{wa}	S_{wa}	DBE_{wa}	$(AI_{mod})_{wa}$	$NOSC_{wa}$
L6	2587	446.000	0.492	1.158	0.183	0.047	10.019	0.300	-0.143
L3	3211	411.239	0.462	1.212	0.285	0.218	8.813	0.276	-0.215
L5	2787	418.643	0.486	1.187	0.223	0.092	9.140	0.287	-0.167
L1	3152	454.038	0.490	1.170	0.215	0.045	10.028	0.293	-0.152
L7	3121	461.399	0.491	1.163	0.162	0.038	10.223	0.296	-0.152
L4	3182	459.733	0.492	1.160	0.160	0.035	10.227	0.298	-0.149
L2	3132	461.720	0.493	1.158	0.152	0.035	10.296	0.298	-0.146

UHs	Formulas	Mw_{wa}	O/C_{wa}	H/C_{wa}	N_{wa}	S_{wa}	DBE_{wa}	$(AI_{mod})_{wa}$	$NOSC_{wa}$
L6	234	595.714	0.034	0.868	0.564	0.538	25.937	0.586	-0.711
L3	115	464.531	0.026	0.892	0.314	0.869	19.341	0.572	-0.743
L5	119	506.644	0.039	0.893	1.048	1.066	20.594	0.577	-0.648
L1	152	511.725	0.039	0.924	1.334	1.183	20.010	0.566	-0.635
L7	105	455.242	0.040	0.938	1.103	1.215	17.328	0.559	-0.634
L4	93	449.137	0.035	0.940	0.764	1.151	17.131	0.552	-0.695
L2	86	457.559	0.041	0.960	0.911	1.159	17.042	0.544	-0.681

Carbohydrates	Formulas	Mw_{wa}	O/C_{wa}	H/C_{wa}	N_{wa}	S_{wa}	DBE_{wa}	$(AI_{mod})_{wa}$	$NOSC_{wa}$
L6	22	541.084	0.789	1.725	1.790	0.663	4.293	-0.593	0.226
L3	71	464.290	0.770	1.658	1.095	0.526	4.472	-0.414	0.152
L5	30	491.212	0.742	1.671	1.331	0.783	4.361	-0.531	0.112
L1	36	434.427	0.787	1.712	1.468	0.643	3.766	-1.213	0.234
L7	24	473.155	0.744	1.598	1.112	0.232	5.185	-0.280	0.114
L4	21	481.051	0.783	1.665	1.192	0.163	4.550	-0.387	0.142
L2	18	470.643	0.761	1.630	1.305	0.117	4.849	-0.364	0.143

Proteins	Formulas	Mw_{wa}	O/C_{wa}	H/C_{wa}	N_{wa}	S_{wa}	DBE_{wa}	$(AI_{mod})_{wa}$	$NOSC_{wa}$
L6	552	385.422	0.371	1.967	0.082	0.827	1.699	-0.213	-1.112
L3	670	381.134	0.384	1.585	0.133	0.463	4.992	0.053	-0.740
L5	441	373.162	0.433	1.578	0.103	0.302	4.833	0.041	-0.660
L1	441	395.406	0.424	1.564	0.227	0.167	5.315	0.060	-0.662
L7	368	383.716	0.412	1.574	0.155	0.140	5.150	0.065	-0.706
L4	381	385.449	0.412	1.574	0.153	0.128	5.162	0.066	-0.708
L2	382	384.260	0.412	1.567	0.184	0.101	5.237	0.072	-0.699

Lipids	Formulas	Mw_{wa}	O/C_{wa}	H/C_{wa}	N_{wa}	S_{wa}	DBE_{wa}	$(AI_{mod})_{wa}$	$NOSC_{wa}$
L6	47	344.902	0.154	1.658	0.249	0.980	4.442	0.113	-1.212
L3	47	322.041	0.164	1.604	0.131	1.008	4.535	0.134	-1.136
L5	38	337.802	0.146	1.641	0.195	1.145	4.304	0.114	-1.199
L1	28	306.086	0.145	1.627	0.031	1.199	4.158	0.119	-1.186
L7	25	325.429	0.158	1.636	0.138	0.988	4.375	0.122	-1.181
L4	34	314.232	0.157	1.651	0.122	1.089	4.068	0.107	-1.184
L2	26	311.962	0.145	1.629	0.048	1.195	4.199	0.117	-1.188

Table S5. The correlations between DOC and molecular parameters of DOM with different molecular compositions (red color indicates a positive correlation, and green color indicates a negative correlation)

	Formulas	Mw_{wa}	O/C_{wa}	H/C_{wa}	N_{wa}	S_{wa}	DBE_{wa}	$(AI_{mod})_{wa}$	$NOSC_{wa}$
**DOM**	0.8950549	-0.47926	-0.6875	0.957098	0.40927	0.742048	-0.82112	-0.95364	-0.90364
**CAs**	0.783608	0.830327	-0.59049	0.011452	0.560681	0.374771	0.678948	0.410286	-0.54729
**UHs**	0.9265217	0.930404	-0.39547	-0.91875	-0.39229	-0.91552	0.984793	0.907953	-0.36566
**Lignins**	-0.80078	-0.43502	-0.16047	0.153667	0.371474	0.226176	-0.33055	-0.0841	-0.08716
**Tannins**	-0.690691	-0.35381	-0.56062	0.257972	0.120183	0.156568	-0.32961	-0.16435	-0.50948
**Lipids**	0.7748551	0.70998	0.042771	0.245522	0.677149	-0.38088	0.569379	0.029413	-0.30203
**Proteins**	0.6651263	-0.04553	-0.61698	0.856399	-0.61552	0.95125	-0.87604	-0.88747	-0.81354
**Carbohydrates**	0.1813825	0.647359	0.395437	0.760003	0.784483	0.763197	-0.51127	-0.31096	0.609719

Table S6. Molecular parameters of DOM with different elemental compositions.

Location	Element	Formulas	Mw_{wa}	C_{wa}	O/C_{wa}	H/C_{wa}	N/C_{wa}	S/C_{wa}	O/N_{wa}	O/S_{wa}	DBE_{wa}	$(AI_{mod})_{wa}$	$NOSC_{wa}$
L6	CHO	2381	448.038	21.454	0.505	1.141	0.000	0.000	–	–	10.343	0.303	-0.131
	CHON	1701	455.348	21.944	0.478	1.077	0.065	0.000	10.211	–	12.223	0.385	0.073
	CHOS	724	383.265	18.340	0.346	1.886	0.000	0.060	–	1.904	2.715	-0.163	-1.073
	CHONS	456	594.812	31.657	0.211	1.024	0.106	0.048	4.799	9.225	18.247	0.465	-0.188
L3	CHO	1825	423.763	21.018	0.465	1.192	0.000	0.000	–	–	9.786	0.288	-0.263
	CHON	1703	418.719	19.449	0.489	1.130	0.075	0.000	8.999	–	10.220	0.359	0.073
	CHOS	979	380.002	18.655	0.360	1.293	0.000	0.062	–	3.471	7.938	0.210	-0.449
	CHONS	703	421.245	18.658	0.475	1.212	0.085	0.067	11.832	12.879	9.336	0.220	0.126
L5	CHO	2294	415.682	19.798	0.498	1.191	0.000	0.000	–	–	9.023	0.280	-0.195
	CHON	1678	422.106	19.821	0.490	1.078	1.306	0.000	9.627	–	10.989	0.394	0.114
	CHOS	704	388.397	18.328	0.416	1.310	0.000	1.045	–	5.469	7.626	0.165	-0.354
	CHONS	485	529.312	26.885	0.314	1.063	2.333	1.337	11.101	15.506	15.231	0.384	-0.036
L1	CHO	2353	452.129	21.292	0.513	1.167	0.000	0.000	–	–	9.847	0.284	-0.140
	CHON	1776	454.581	21.139	0.507	1.075	1.259	0.000	7.609	–	11.607	0.379	0.131
	CHOS	396	386.939	20.157	0.336	1.173	0.000	1.057	–	4.122	10.038	0.276	-0.385
	CHONS	482	557.680	29.432	0.241	1.047	2.627	1.504	5.714	8.659	17.221	0.369	-0.151
L7	CHO	2527	461.187	21.710	0.514	1.152	0.000	0.000	–	–	10.183	0.292	-0.123
	CHON	1484	446.326	20.211	0.519	1.094	0.064	0.000	10.059	–	10.761	0.363	0.135
	CHOS	393	385.083	20.511	0.314	1.172	0.000	0.056	–	4.374	10.223	0.291	-0.432
	CHONS	271	508.510	27.318	0.179	1.003	0.116	0.057	3.996	6.819	16.303	0.510	-0.185
L4	CHO	2574	459.246	21.633	0.513	1.151	0.000	0.000	–	–	10.166	0.294	-0.124
	CHON	1523	445.105	20.073	0.524	1.098	0.063	0.000	9.929	–	10.633	0.358	0.138
	CHOS	399	387.893	21.148	0.289	1.159	0.000	0.055	–	4.092	10.689	0.309	-0.471
	CHONS	218	510.460	27.818	0.171	0.978	0.111	0.055	3.980	6.307	16.934	0.526	-0.194
L2	CHO	2568	461.605	21.717	0.515	1.146	0.000	0.000	–	–	10.263	0.296	-0.115
	CHON	1507	447.567	20.229	0.522	1.097	0.062	0.000	10.011	–	10.742	0.358	0.134
	CHOS	379	387.880	20.712	0.311	1.143	0.000	0.057	–	4.503	10.592	0.308	-0.407
	CHONS	229	510.018	27.441	0.188	0.982	0.112	0.056	4.359	6.965	16.698	0.515	-0.156

Table S7. The correlations between DOC and molecular parameters of DOM with different elemental compositions (red color indicates a positive correlation, and green color indicates a negative correlation).

	Formulas	Mw_{wa}	O/C_{wa}	H/C_{wa}	N/C_{wa}	S/C_{wa}	DBE_{wa}	$(AI_{mod})_{wa}$	$NOSC_{wa}$
CHO	-0.406717	-0.42465	-0.33564	0.062003	#DIV/0!	#DIV/0!	-0.06957	0.242462	-0.25516
CHON	0.7057891	0.033714	-0.92787	-0.2878	0.111669	#DIV/0!	0.711521	0.656787	-0.83313
CHOS	0.6341953	-0.47803	0.515969	0.932299	#DIV/0!	0.11092	-0.96405	-0.94978	-0.79543
CHONS	0.5971864	0.450818	0.288308	0.332347	0.099455	0.09998	0.029465	-0.34792	0.15895

References

1. Stubbins, A.; Spencer, R. G. M.; Chen, H.; Hatcher, P. G.; Mopper, K.; Hernes, P. J.; Mwamba, V. L.; Mangangu, A. M.; Wabakanghanzi, J. N.; Six, J., Illuminated darkness: Molecular signatures of Congo River dissolved organic matter and its photochemical alteration as revealed by ultrahigh precision mass spectrometry. *Limnology and Oceanography* **2010,** *55*, (4), 1467-1477.
2. Wang, X.; He, C.; Shi, Q.; Song, G., Molecular characterization by ultrahigh resolution mass spectrometry of dissolved black carbon-like molecules in summer along the Pearl River Estuary, China. *Environmental Advances* **2022,** *9*.
3. Zhao, P.; Du, Z.; Fu, Q.; Ai, J.; Hu, A.; Wang, D.; Zhang, W., Molecular composition and chemodiversity of dissolved organic matter in wastewater sludge via Fourier transform ion cyclotron resonance mass spectrometry: Effects of extraction methods and electrospray ionization modes. *Water Res* **2023,** *232*, 119687.
4. He, C.; He, D.; Chen, C.; Shi, Q., Application of Fourier transform ion cyclotron resonance mass spectrometry in molecular characterization of dissolved organic matter. *Science China Earth Sciences* **2022,** *65*, (12), 2219-2236.
5. Zhao, Y.; Huang, Y.; Sun, X.; Dong, G.; Li, Y.; Ma, M., Forest Fire Mapping Using Multi-Source Remote Sensing Data: A Case Study in Chongqing. *Remote Sensing* **2023,** *15*, (9).
